# A Pulmonary Vascular Model From Endothelialized Whole Organ Scaffolds

**DOI:** 10.3389/fbioe.2021.760309

**Published:** 2021-11-19

**Authors:** Yifan Yuan, Katherine L. Leiby, Allison M. Greaney, Micha Sam Brickman Raredon, Hong Qian, Jonas C. Schupp, Alexander J. Engler, Pavlina Baevova, Taylor S. Adams, Mehmet H. Kural, Juan Wang, Tomohiro Obata, Mervin C. Yoder, Naftali Kaminski, Laura E. Niklason

**Affiliations:** ^1^ Vascular Biology and Therapeutics Program, Yale University School of Medicine, New Haven, CT, United States; ^2^ Department of Anesthesiology, Yale University, New Haven, CT, United States; ^3^ Department of Biomedical Engineering, Yale University, New Haven, CT, United States; ^4^ Medical Scientist Training Program, Yale University, New Haven, CT, United States; ^5^ Section of Pulmonary, Critical Care, and Sleep Medicine, Yale School of Medicine, New Haven, CT, United States; ^6^ Department of Respiratory Medicine, Hannover Medical School and Biomedical Research in End-stage and Obstructive Lung Disease Hannover, German Lung Research Center (DZL), Hannover, Germany; ^7^ Indiana Center for Regenerative Medicine and Engineering, Indiana University School of Medicine, Indianapolis, IN, United States

**Keywords:** whole lung tissue engineering, endothelium, pulmonary vasculature, *in vitro* disease modeling, single-cell RNA-sequencing

## Abstract

The development of an *in vitro* system for the study of lung vascular disease is critical to understanding human pathologies. Conventional culture systems fail to fully recapitulate native microenvironmental conditions and are typically limited in their ability to represent human pathophysiology for the study of disease and drug mechanisms. Whole organ decellularization provides a means to developing a construct that recapitulates structural, mechanical, and biological features of a complete vascular structure. Here, we developed a culture protocol to improve endothelial cell coverage in whole lung scaffolds and used single-cell RNA-sequencing analysis to explore the impact of decellularized whole lung scaffolds on endothelial phenotypes and functions in a biomimetic bioreactor system. Intriguingly, we found that the phenotype and functional signals of primary pulmonary microvascular revert back—at least partially—toward native lung endothelium. Additionally, human induced pluripotent stem cell-derived endothelium cultured in decellularized lung systems start to gain various native human endothelial phenotypes. Vascular barrier function was partially restored, while small capillaries remained patent in endothelial cell-repopulated lungs. To evaluate the ability of the engineered endothelium to modulate permeability in response to exogenous stimuli, lipopolysaccharide (LPS) was introduced into repopulated lungs to simulate acute lung injury. After LPS treatment, proinflammatory signals were significantly increased and the vascular barrier was impaired. Taken together, these results demonstrate a novel platform that recapitulates some pulmonary microvascular functions and phenotypes at a whole organ level. This development may help pave the way for using the whole organ engineering approach to model vascular diseases.

## 1 Introduction

Diseases of the lung vasculature, including primary pulmonary hypertension, capillary leak syndrome/acute respiratory distress syndrome (ARDS), inhalation injury, and the novel coronavirus disease-19 (COVID-19), are difficult to study *in vitro* due to the lack of functional *ex vivo* models (Huertas et al., 2018; Ackermann et al., 2020). Typical culture systems do not recapitulate lung macro- and micro-vascular structure, and the phenotype of cultured endothelium on plastic differs dramatically from that of native (Pampaloni et al., 2007; Schupp et al., 2020; Schupp et al., 2021). Pulmonary vascular diseases can be characterized by remodeling and loss of microvessels, processes which are often attributed to pathological responses in vascular endothelium (Mélot and Naeije, 2011).

Vascular niche characteristics, such as paracrine factors, hemodynamics, extracellular matrix (ECM), and cell-cell crosstalk, are crucial for regulating endothelial maturation and maintaining vascular homeostasis. Recently, organoid and microfluidics-based *in vitro* culture systems have emerged as tools for modeling vascular diseases (Tsai et al., 2012; Qiu et al., 2018; Wimmer et al., 2019; Monteil et al., 2020). These systems typically involve culturing differentiated or mature endothelial cells in polydimethylsiloxane (PDMS) or hydrogel-based structures under some degree of perfusion (Tsai et al., 2012; Qiu et al., 2018; Wimmer et al., 2019). However, these systems only recapitulate a very few physiological properties, such as shear stress, while failing to mimic important other aspects of organ physiology such as appropriate substrate stiffness, physiological mechanical stretch, and co-culture with other cell types of the airway or alveolar niche (Tsai et al., 2012; Qiu et al., 2018). Further, microfluidic systems fail to reconstitute the structural, compositional, and biological features of native ECM, which are critical for intracellular regulation within the vascular compartment. Native lung tissues derived from humans or animals that are cultured as whole organs *ex vivo* rapidly lose cell viability and barrier function during organ culture, and cannot be maintained in a reproducible way for extended time periods. As a result, no *in vitro* platform exists that can simulate native pulmonary vascular phenotypes and functions for long time periods and in a controlled fashion.

Whole organ decellularization opens a door to developing a construct that can recapitulate a complete vascular structure. Unlike native whole organs, acellular matrices can be repopulated with controlled cell numbers and cell types, and cellular viability can be maintained for many days since cells accommodate to the *in vitro* environment. But while repopulation of acellular lung matrices has been reported by several groups (Petersen et al., 2010; Ren et al., 2015; Le et al., 2017), evaluation of endothelial and microvascular functionality and phenotypes in these model systems has heretofore been fairly limited.

Protocols for the characterization of decellularized lung scaffolds have been published previously (Balestrini et al., 2016; Calle et al., 2016). ECM components remaining after decellularization in rat, and human lungs have been examined by quantitative proteomics, showing that a decellularization technique employing Triton detergent and sodium deoxycholate (SDC) results in an intact vascular architecture and near-native retention of laminins, proteoglycans, and other basement membrane and ECM-associated proteins (Calle et al., 2016; Li et al., 2016). Additionally, the decellularized matrix itself immobilizes soluble and insoluble cues, including VEGF, FGF, BMP, HGF, and PDGF that drive pulmonary cell regeneration (Booth et al., 2012; Gilpin et al., 2014; Ullah et al., 2019). Therefore, the decellularized lung scaffold represents a potential platform to recapitulate physiological niches in the lung parenchyma, by virtue of retained matrix protein composition, soluble and insoluble cues, 3-dimensional structure, and mechanical properties.

**FIGURE 1:**
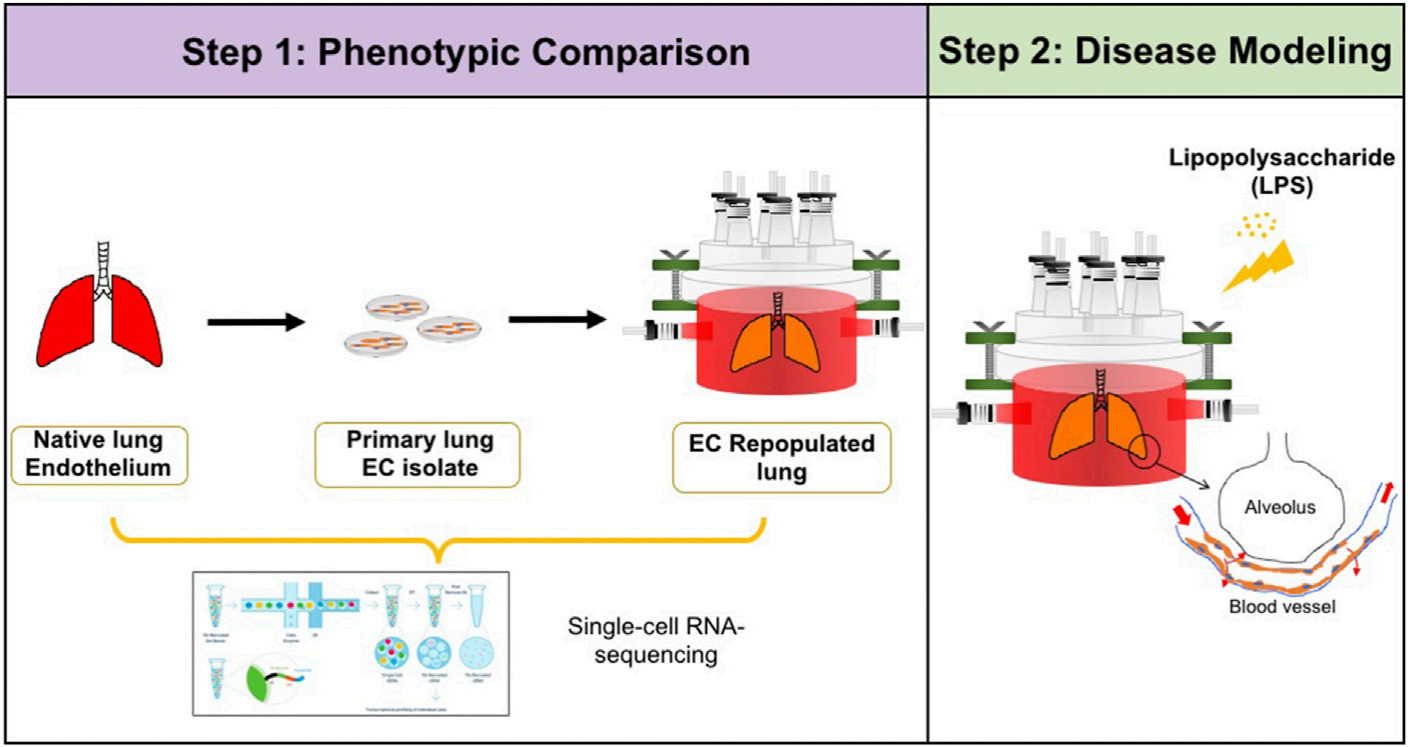
Schematic of experimental design. Step 1: single-cell RNA-sequencing analysis was performed to compare the phenotypic and functional signals among native pulmonary endothelium, primary pulmonary endothelium cultured on tissue culture plastic, and in whole lung scaffolds. Step 2: Lipopolysaccharide (LPS) was introduced into endothelialized whole lung scaffolds to simulate inflammation.

In this study, we explored the impact of decellularized whole lung scaffolds on endothelial cell phenotype and functions in the biomimetic bioreactor system (Supplementary Figure S1). As evaluated by single cell RNA sequencing (scRNAseq), primary rodent pulmonary microvascular or human induced pluripotent stem cell-derived endothelium showed a dramatic shift back to phenotypes resembling native after transitioning from expansion on tissue culture plates, into the lung matrix scaffold. After introduction of lipopolysaccharide (LPS) into endothelial-repopulated lung matrices, the endothelium was actively responsive, showing increased pro-inflammatory and matrix degradation characteristics, and decreased barrier function at the whole organ level, similar to the effect in native lungs (Figure 1). Thus, this study shows the potential of using the bioengineered whole organ as a platform for pulmonary vascular disease modeling.

## 2 Results

### 2.1 Reendothelialization of Decellularized Whole Lung Scaffolds

The isolation of pulmonary microvascular endothelial cells (PMECs) was performed as previously described (Alphonse et al., 2015). Endothelial cells were isolated from the distal parenchyma of the lung in postnatal day 7 rats by CD31-labeled magnetic bead selection (Supplementary Figure S2A). PMECs became morphologically homogenous as early as passage 1, with no evident mesenchyme contamination (data not shown). Flow cytometry data confirmed that PMECs at passage 1 contain 99.5 ± 0.3% CD31 and 99.6 ± 0.2% CD144 positive cells while having low or no expression of CD90 and CD45 (Supplementary Figure S2B). These PMECs were rapidly proliferative—with an average doubling time of ∼26.6 h across passages—and displayed key endothelial characteristics including expression of CD31, vWF, VE-Cadherin, eNOS, and Griffonia simplicifolia lectin, as well as acLDL uptake (Supplementary Figures S2C,D).

We cultured PMECs in decellularized lung scaffolds in a biomimetic bioreactor, as previously described (Yuan et al., 2019; Engler et al., 2019). Acellular lung scaffolds were produced by decellularizing adult native rat lungs using Triton and SDC detergents, followed by copious washing with PBS and sterilization with antibiotics. Lung scaffolds were then seeded with 50 million P4 PMECs at a concentration of 250,000 cells/mL into the pulmonary vein, followed by seeding another 50 million cells into the pulmonary artery. Cultures were maintained with a flow of medium delivered via the pulmonary artery under laminar flow. The trachea and pulmonary vein were cannulated to monitor real-time fluid flows and pressures during the culture process.

Seeding of cultured endothelial cells into acellular organ microvasculature has proven difficult in prior studies, in part because the cytoplasmic volume of cultured endothelium is much larger than that of native cells (Pugacheva et al., 2006). Prior studies have shown that cultured EC can lodge in arteriolar and venular structures immediately after seeding into the pulmonary vasculature (Le et al., 2017). The physically large cell bodies may be blocked from ingress into small decellularized capillary lumens having diameters of 5–8 microns or less (Townsley, 2012). We therefore investigated whether an exogenous agent that can impact EC cytoplasmic organization could affect the ability of cells to transit or migrate into small capillary lumens. Rho-associated protein kinases (ROCK) inhibitor (Y27632) can re-organize the actin cytoskeleton, and further remodel overall cell morphology (Amano et al., 2010). Additionally, ROCK inhibition could potentially provide pro-survival signals to support EC survival and growth in decellularized lung scaffolds (Watanabe et al., 2007). We therefore applied 10 μM Y27632 to endothelial-containing lung cultures for 7 days, to determine the impact on endothelial repopulation and cell spreading in the lung matrix.

**FIGURE 2:**
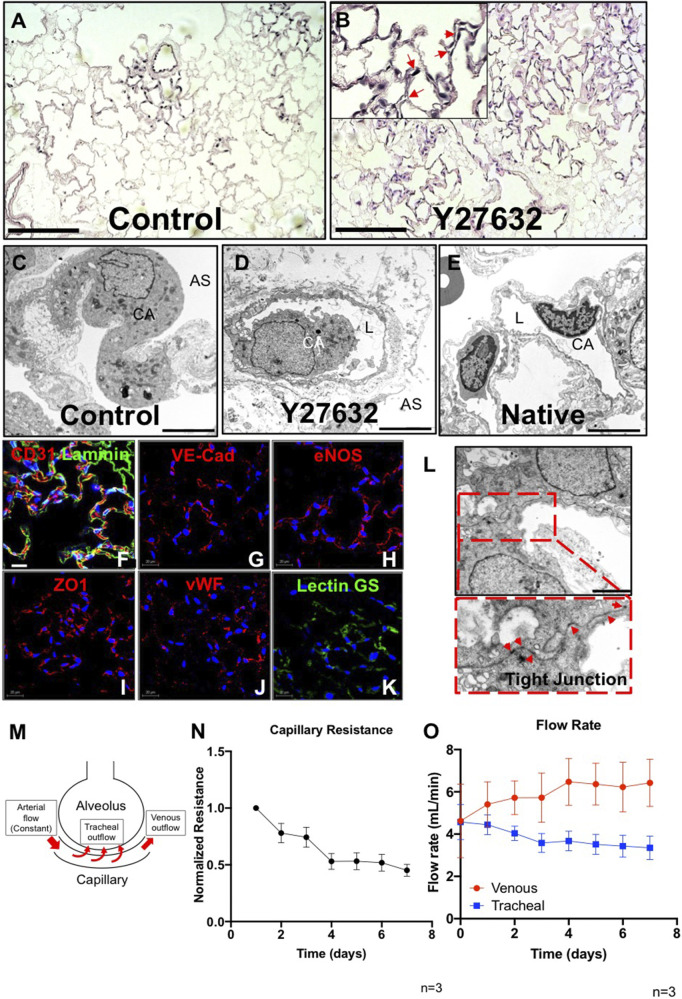
Characterization of PMEC-repopulated lungs. **(A,B)** H/E image of a representative section in control **(A)** and Y27632-treated repopulated lungs **(B)**. Scale bars 150 μm, arrows point to reconstituted capillary lumens. **(C,D,E)** TEM images of control, Y27632-treated repopulated lungs, and native lungs, respectively. Scale bars 5 μm, CA: Capillary; AS: Alveolar Space; L: Lumen. **(F–K)** Immunostaining for CD31, VE-Cad, eNOS, ZO1, vWF, and lectin GS in repopulated lungs, scale bar 20 μm. **(L)** Representative TEM image of tight junctions in repopulated lungs. Arrowheads indicate tight junctions, scale bar 2 μm. **(M)** Schematic description of the flow paths in decellularized lung vasculature. **(N,O)** Non-invasive measurements were performed daily to assess the mechanical characteristics in repopulated lungs. **(N)** Capillary resistances to fluid flow within the vascular bed, versus time. **(O)** Flow rates: venous (red), and tracheal (blue) outflows versus time. “n” indicates experimental replicates. There were three independent experiments performed for each condition.

In control lungs, cells had a round morphology, with limited repopulation throughout the lung vasculature especially in microvasculature, and no or minimal patent lumens were visible (Figure 2A). Interestingly, after treatment with Y27632, PMECs appeared to repopulate extensively throughout the large and small vasculature of the lung matrix (Supplementary Figure S3A). Endothelial cells had a native-like and flattened morphology in the vasculature, and also formed observable patent lumens in the small vessels throughout the repopulated organs (Figure 2B). Consistently, transmission electron microscopy (TEM) images of the repopulated lungs cultured with Y27632 treatment show patent lumens in the alveolar-capillary network, with flattened cytoplasmic processes covering the inner surface of the vessel, similar to those seen in native lungs (Figures 2D,E), whereas such lumens were not present under control conditions (Figure 2C). Immunostaining for PECAM1, Laminin, VE-Cadherin, vWF, eNOS, ZO1, and Lectin GS confirmed that PMECs remained inside of the vasculature and maintained their expression of endothelial markers after 7 days of lung culture (Figures 2F–K). Additionally, representative TEM images demonstrate the formation of tight junctions between cells, suggesting that the vascular barrier is starting to reconstruct in our repopulated lungs (Figure 2L). Since the repopulated lungs without ROCK inhibitor have limited EC repopulation throughout the organ, we have therefore only analyzed the vascular functions and transcriptomic information in lungs supplied with ROCK inhibitor in subsequent studies.

To determine whether PMEC could establish measurable barrier function within the repopulated lungs, we applied a non-invasive mechanical measurement that was previously developed (Engler et al., 2019) to monitor the pressure changes generated by fluid flowing across the alveolar basement membrane (from the repopulated capillaries, into the alveolar air sacs (tracheal outflow) and within the vasculature (venous outflow) (Figure 2M). These non-invasive pressure measurements allow the inference of alveolar barrier function during culture. Measurement of average internal pressures (capillary entry pressure, and capillary exit pressure) is accomplished by briefly shutting off flow into or out of the organ, and measuring transient pressures at the end-organ level using piezoelectric pressure sensors situated within the bioreactor system (Engler et al., 2019).

In endothelial repopulated lungs, measurements of pressures, and of fluid translocation across the alveolar membrane and exiting the trachea, revealed that capillary resistances to fluid flow through the vascular bed gradually decreased over the 7-days culture period (Figure 2N). This may indicate that endothelial cells, which initially occlude arterioles and venules at the time of cell seeding (Supplementary Figure S3B), may migrate into capillaries and form patent lumens that can then conduct vascular flow, while increasing overall coverage throughout the vasculature. Evidence to support this supposition is seen in H&E histology (Figure 2B; Supplementary Figure S3A). Measurement of fluid translocation over time indicates that there are increasing trends of outflows in the pulmonary vein, indicating increasing flow rate exiting the vasculature (as opposed to leaking across the alveolar barrier and into the airways) over time. Similarly, flow exiting the trachea, which is indicative of net fluid leak across the alveolar barrier, decreased over time in endothelial-repopulated lungs, implying functional barrier formation by cultured PMECs (Figure 2O). Since repopulated lungs lack alveolar epithelium, which is a significant component of native physiological barrier, we did not anticipate a full reconstitution of native levels of alveolar barrier. However, the ability to detect some barrier function conferred by cultured endothelium is a reflection of functional competency of the cells. In here, we have optimized our protocol by adding ROCK inhibitor and determined that the cultured endothelium could form a functional monolayer that partially reconstructs vascular barrier function in whole lung scaffolds.

### 2.2 PMECs Cultured in Lung Scaffolds Regain Native-Like Functional Endothelial Character Compared to PMEC on Tissue Culture Plastic

**FIGURE 3:**
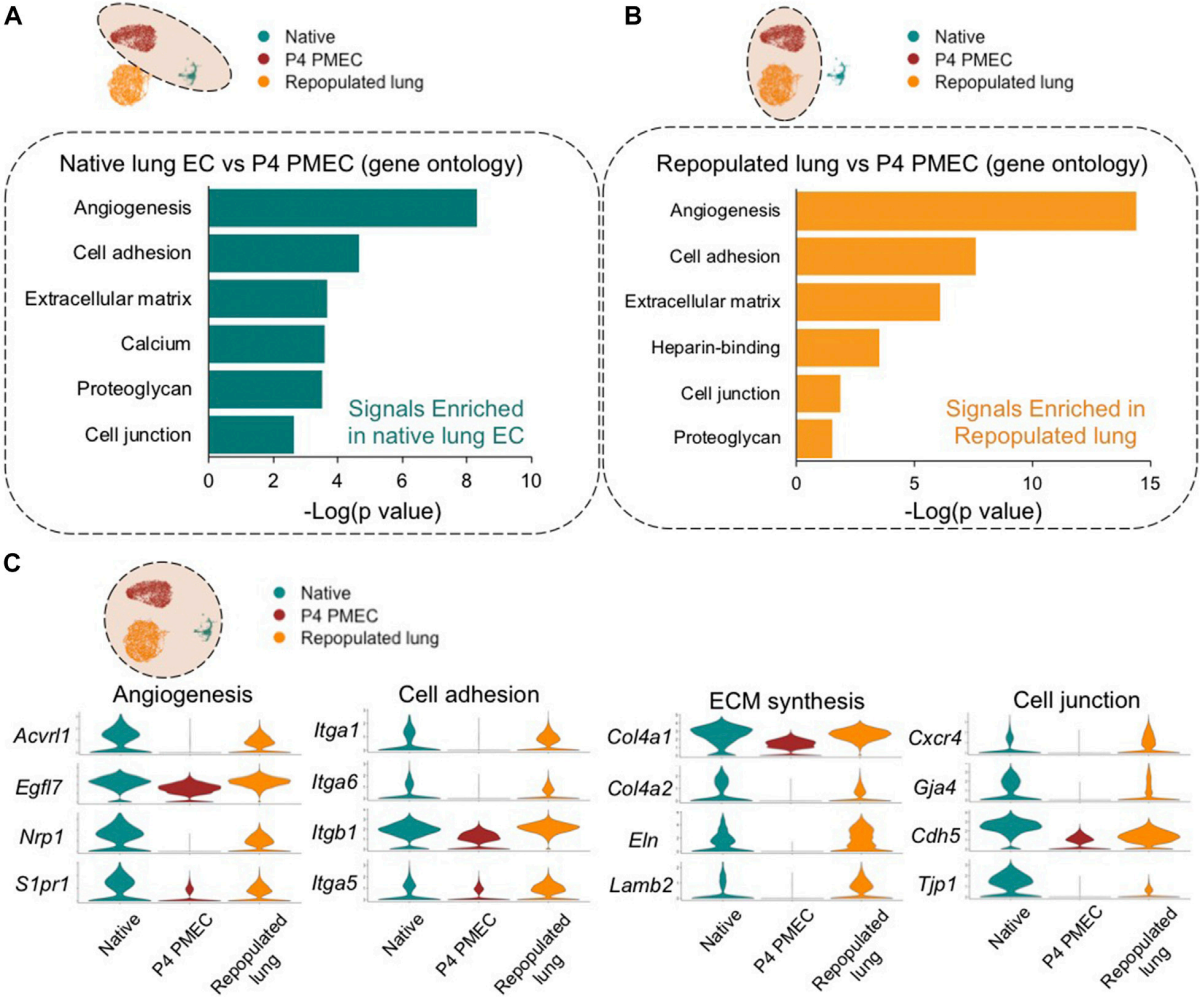
Global comparison amongst Native, P4 PMEC, and Repopulated lung. **(A,B)** Gene ontology analysis shows the top upregulated signals based on the top DEGs of comparisons between Native endothelium and P4 PMEC **(A)**, and between Repopulated lung and P4 PMEC **(B)**. **(C)** VlnPlots of representative genes of angiogenesis, cell adhesion, ECM synthesis, and cell junction in the merged dataset of Native, P4 PMEC, and Repopulated lung samples.

To understand the evolving phenotype of PMECs in lung scaffolds, postnatal day 7 rodent native whole lung tissues (13,619 cells), pre-seeded PMECs grown in tissue culture flasks (9,321 cells), and PMEC-repopulated engineered lungs (6,188 cells) were processed through single-cell transcriptomic analysis. Whole lung tissues were harvested from three male and three female rats, and the native endothelial populations were sub-clustered by selecting for VE-cadherin (*Cdh5*) positive cells while excluding cells expressing *Col1a1* (mesenchyme), *Epcam* (epithelium), and *Ptprc* (leukocyte). We then merged, scaled, and compared the transcriptomic information among native lung endothelium, pre-seeded PMECs, and PMEC-repopulated lungs datasets. The gene ontology analysis (DAVID) of the top differentially expressed genes (DEGs) between native endothelium and cultured PMECs revealed that during expansion, PMECs markedly lost native molecular signals that are related to angiogenesis (e.g., *Acvrl1*, *Nrp1*, and *Egfl7*) (Nichol and Stuhlmann, 2012; Kofler and Simons, 2015; Rochon et al., 2016), cell adhesion (e.g., *Itgb1*, *Fbln5*, and *Itga6*) (Preis et al., 2006), ECM synthesis (e.g., *Col15a1*, *Eln*, *Col4a1*, and *Lamb2*), and cell junctions (*Cxcr4*, *Cldn5*, and *Prx*) (Wang et al., 2018) (Figure 3A; Supplementary Table S1). After culturing in acellular whole lung scaffolds for 7 days under arterial perfusion, intriguingly, many of the molecular signals that are related to angiogenesis, cell adhesion, cell junctions, and ECM synthesis were increased (Figure 3B; Supplementary Table S2). Many of the increased genes are associated with angiogenesis, cell adhesion, ECM synthesis, and cell junctions, including *Acvrl1*, *Nrp1*, *Egfl7*, *Itga6*, *Itga3*, *Col4a1*, *Eln*, *Cxcr4*, and *Gja4* (Figure 3C). Consistent with the findings from scRNAseq, the levels of Itga6, Itgb1, Acvrl1, Eln, and Col4a1 evaluated by western blotting or qRT-PCR were significantly increased in cells extracted from repopulated lungs, as compared to pre-seeded PMECs, across at least three independent experiments (Supplementary Figure S4). We have additionally studied the expression level of glycocalyx-associated genes such as syndecan, glypican, perlecan, and biglycan in ECs before and after culturing in lung scaffolds. Only biglycan (gene name: bgn) was found to be higher in repopulated lung as compared to pre-seeded cells (Supplementary Table S2). Hence, the global comparisons among PMEC before and after culturing in lung scaffolds, and native lung endothelium, suggest that the microenvironmental cues in lung scaffolds, including ECM components and structure, insoluble or soluble cues, and shear stress, could potentially induce the endothelial cells to regain some aspects of the native functional phenotype that is lost during *in vitro* expansion on tissue culture plastic.

### 2.3 PMECs Partially Regain Native Heterogeneous Endothelial Phenotypes in Repopulated Lungs

**FIGURE 4:**
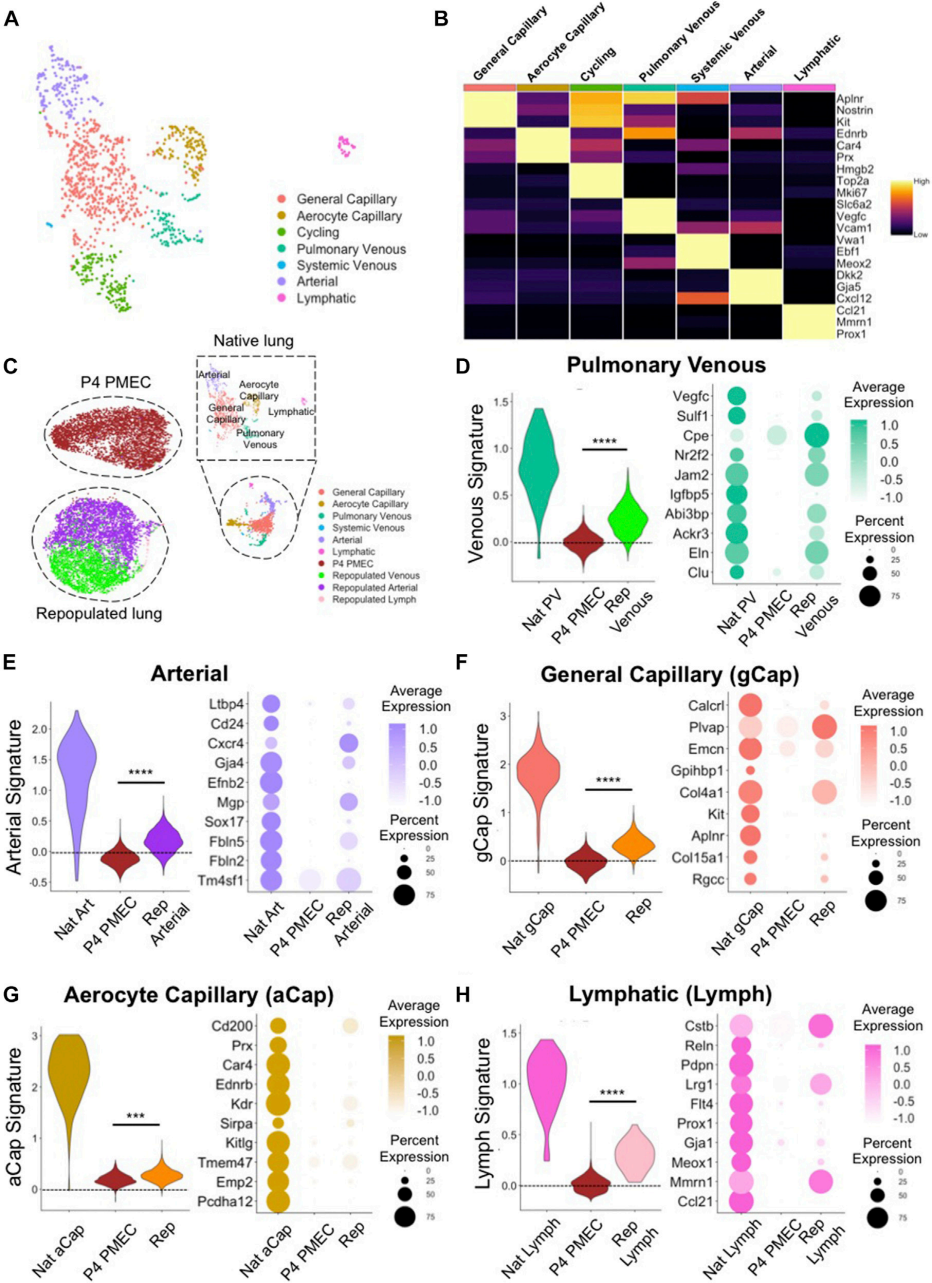
scRNAseq evaluation of native and engineered rat pulmonary endothelium. **(A)** Uniform Manifold Approximation and Projection (UMAP) of the native pulmonary endothelial clusters. **(B)** Heatmap of top 3 DEGs for each cluster. **(C)** UMAPplots of merged object of native pulmonary endothelial clusters, pre-seeded PMECs, and repopulated lungs. **(D–H)** Dotplots and Module scores for **(D)** pulmonary venous, **(E)** arterial, **(F)** gCap, **(G)** aCap, and **(H)** lymph endothelium amongst native and engineered endothelium. Dot plots show the comparison for top 10 select markers of each native lung EC subtype. Samples were merged and re-scaled. Module scores were calculated based on the top 20 DEGs of each native lung EC subtypes in each engineered EC dataset at single cell level. Violin plots represent the distribution of module score for each EC subtype among native endothelium, P4 PMEC, and repopulated lung datasets. Scores >0 indicate enriched expression, compared to random control gene sets. ***, and **** indicate *p* < 0.001, and *p* < 0.0001, respectively.

To characterize heterogenous endothelial phenotypes expressed within PMECs before and after culturing in lung scaffolds, we first determined the molecular signatures of the various endothelial phenotypes present in native lungs. Based on top DEGs, we identified 7 major endothelial clusters in native rat lung at postnatal day 7: general capillary (gCap); aerocyte capillary (aCap); arterial; pulmonary venous; systemic venous; cycling; and lymphatic (Lymph) endothelium (Figure 4A). The general capillary (gCap), aerocyte capillary (aCap), arterial, pulmonary venous, systemic venous, cycling, and lymphatic clusters expressed canonical endothelial markers of their respective sub-types, including *Aplnr*, and *Kit* for gCap (Kalucka et al., 2020; Travaglini et al., 2020); *Car4* and *Prx* for aCap (Gillich et al., 2020; Kalucka et al., 2020; Travaglini et al., 2020); *Gja5*, and *Cxcl12* for arterial ECs (Kalucka et al., 2020; Travaglini et al., 2020); *Vegfc*, and *Vcam1* for pulmonary venous ECs (Kalucka et al., 2020; Travaglini et al., 2020); *Vwa1*, and *Ebf1* for systemic venous ECs (Schupp et al., 2020); *Mki67*, and *Top2a* for cycling ECs; *Prox1*, and *Ccl21* for lymph ECs (Kalucka et al., 2020; Travaglini et al., 2020) (Figure 4B). Since the systemic venous EC cluster is a tiny population in native rodent lung (Verloop, 1949; Parent, 2015), it was excluded in further comparisons. To confirm protein expression in various native EC populations, we selected two specific genes amongst the DEGs for each cell type. Immunostaining of native rat lung shows protein expression of Connexin 40 (*Gja5*) and Cxcl12 in arterial ECs; Vcam1 and Coup-TFII (*Nr2f2*) in venous ECs; and Aplnr, Kit, Prx, and Car4 in small capillaries, but not in large vessels (Supplementary Figure S5). Taken together, the scRNAseq analysis and the immunostaining of whole lung tissues confirm that endothelium in rodent pulmonary vasculature exhibits molecular heterogeneity that is consistent with recent published reports (Schupp et al., 2020; Travaglini et al., 2020; Schupp et al., 2021).

To examine the similarities and differences between engineered and native samples, we applied the “Merge” function in Seurat and combined native lung endothelium, P4 PMECs (pre-seeded and grown on tissue culture plastic), and PMEC-repopulated lungs into a single dataset. P4 PMECs were a homogenous endothelial population, with an overall phenotype quite different from that of any of the native endothelial clusters observed in this study. In contrast, and unlike the pre-seeded PMEC population, the ECs in repopulated lungs were a mixture of arterial-like, venous-like, and also a small population of lymph-like endothelium (Supplementary Figure S6). Arterial-like cells (Repopulated Arterial) expressed canonical arterial (e.g., *Cxcr4*, *Gja4*, and *Kcne3*) markers (Su et al., 2018; Kalucka et al., 2020; Schupp et al., 2020). In contrast, venous-like endothelium (Repopulated Venous) expressed venous (e.g., *Ackr3*, *Nr2f2*, and *Jam2*) markers (You et al., 2005; dela Paz and D’Amore, 2009; Schupp et al., 2020). A small population of lymph-like endothelium (Repopulated Lymph) expressed lymph marker (e.g., *Prox1*) (Wigle et al., 2002) (Supplementary Figure S6).

We further used “Addmodulescore” function in Seurat and calculated module scores for each cell type/cluster amongst different datasets, using the top 20 DEGs identified from each native lung EC subtype (Supplementary Table S3). Cells in the venous cluster in the repopulated lungs (Repopulated Venous) expressed higher levels of markers for native venous endothelium, such as *Nr2f2*, *Cpe* (Schupp et al., 2020), *Ackr3* (Schupp et al., 2020), and *Eln*, and received significantly higher scores for gene signatures of pulmonary venous endothelium, as compared to pre-seeded PMEC (Figure 4D). Similarly, cells in the arterial cluster in repopulated lungs expressed higher levels of markers for native arterial endothelium *Cxcr4*, *Fbln5* (Noda et al., 2013), *Cd24*, *Gja4*, *Efnb2* (Su et al., 2018), and *Sox17* (Corada et al., 2013), and received significantly higher scores for gene signatures of native arterial endothelium, as compared to pre-seeded cells (Figure 4E). Immunostaining confirmed the protein presence of Fbln5, Eln, and Nr2f2 in repopulated lungs (Supplementary Figure S7B).

Interestingly, repopulated lungs also expressed many specific markers for gCap endothelium (e.g., *Aplnr*, *Col4a1*, *Plvap*, *Rgcc*, and *Col15a1*) (Gillich et al., 2020; Kalucka et al., 2020; Schupp et al., 2020; Travaglini et al., 2020) and for aCap endothelium (e.g., *Kdr*, *Sirpa*, and *Ednrb*) (Gillich et al., 2020; Schupp et al., 2020; Travaglini et al., 2020), and received significantly higher scores for gene signatures of both native cell subtypes, as compared to cells cultured on tissue culture plastic (Figures 4F,G). Furthermore, there are some native capillary endothelial markers which appear to be completely silenced during EC growth on plastic (e.g., *Aplnr*, *Rgcc*, *Ednrb*, and *Sirpa*), that are regained after culture on the decellularized lung matrix (Figures 4F,G). Immunostaining for these previously silenced markers, including Sirpa, and Ednrb, confirmed their presence in repopulated lungs (Supplementary Figure S7B). While expression of these key genes was not fully restored to native levels in engineered lungs, these trends imply that functional pulmonary endothelial phenotypes are supported and encouraged by a physiological lung matrix substrate and biomimetic bioreactor culture conditions.

There was a tiny, but detectable, population of lymphatic endothelium in EC-repopulated lungs that expressed high level of lymphatic markers (e.g., *Mmnr1*, and *Prox1*), suggesting either a leftover of lymphatic cells and progenitors from the starting passaged population, or a limited differentiation effect of the lung matrix scaffold, since all of the endothelium was seeded into the blood, rather than into the lymphatic, vasculature (Figure 4H). Consistent with the findings from scRNAseq, the levels of *Gja4*, *Fbln5*, *Nr2f2*, *Eln*, *Aplnr*, *Rgcc*, *Ednrb*, and *Sirpa*, as evaluated by qRT-PCR were significantly increased in cells from three independent repopulated lungs as compared to pre-seeded PMECs (Supplementary Figure S7A). These pairwise comparisons indicate that PMECs are changing phenotypes to more resemble the native complement of ECs when cultured in the decellularized lung scaffolds in a bioreactor system.

### 2.4 Human iPSC-ECFCs Start to Gain Human Lung Endothelial Phenotypes in Repopulated Lungs

**FIGURE 5:**
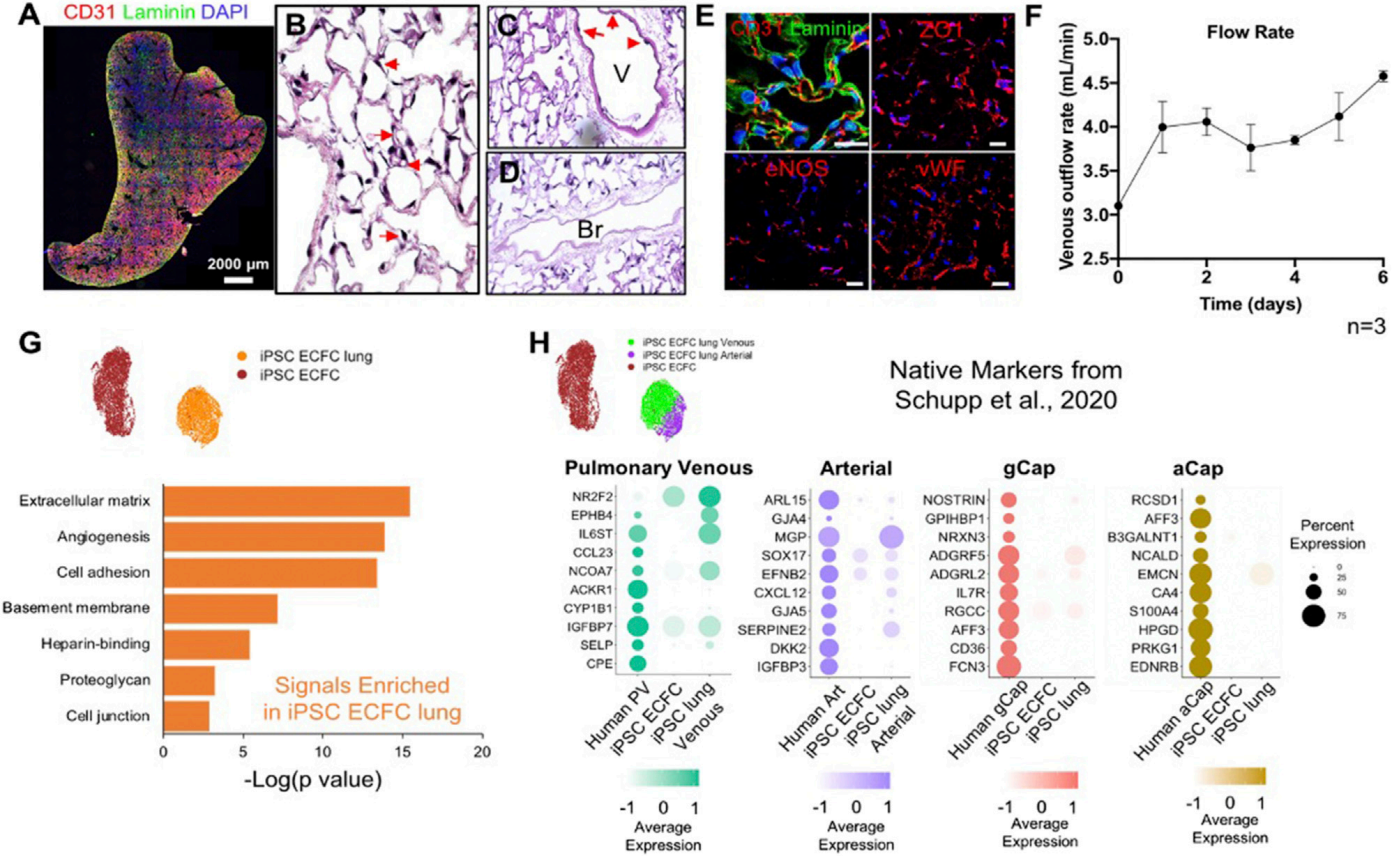
Human iPSC-ECFCs start to gain native EC phenotype in repopulated lungs. **(A)** Immunostaining of CD31 and Laminin in iPSC-ECFC-repopulated lungs. Scale bar 2,000 μm. **(B–D)** H/E image of a representative section iPSC-ECFC repopulated lungs. Arrows in **(B)** point to reconstituted capillary lumens; arrows in **(C)** point to endothelial cells in the vasculature but not in bronchia. V: Blood Vessel; Br: Bronchi **(E)** Immunostaining for CD31, laminin, ZO1, eNOS, and vWF in repopulated lungs, scale bar 20 μm. **(F)** Venous outflows versus time, *n* = 3. **(G)** Gene ontology analysis of the top DEGs (*p* < 0.05) enriched in iPSC-ECFC lungs as compared to pre-seeded iPSC-ECFCs. **(H)** Dot plots show the comparison for top 10 select native markers of human pulmonary venous, arterial, gCap, and aCap amongst native human lung endothelium, pre-seeded iPSC-ECFCs, and iPSC-ECFC-repopulated lungs. Samples were merged and re-scaled.

To develop a modeling system to study pulmonary vascular diseases, we explored the phenotypic changes of human induced pluripotent stem cell-derived endothelial colony-forming cells (iPSC-ECFCs) in acellular whole lung scaffolds. We seeded 25 million iPSC-ECFCs into the pulmonary vein, followed by seeding another 25 million cells into the pulmonary artery. Cultures were maintained with a flow of medium delivered via the pulmonary artery. After culturing human iPSC-ECFCs in the decellularized lungs with Y27632 for 6 days, endothelial cells appeared to repopulate extensively throughout the lung vasculature (Figure 5A), while patent vascular lumens were appreciable (Figure 5B). The iPSC-ECFCs maintained their endothelial phenotype within lung scaffolds, as evidenced by expression of CD31, ZO1, eNOS, and vWF (Figure 5E). The venous outflows continuously increased over time (Figure 5F), suggesting an increased barrier formation in iPSC-ECFC repopulated lungs. From these data it appears that under current culture conditions, both rodent primary PMECs and human iPSC-ECFCs express endothelial-specific markers in repopulated lung matrix, and, over time, contribute to alveolar barrier formation in culture. Combined with observable lumen formation and decreased vascular flow resistance during culture, this implies the reconstitution of a highly functional endothelial lining within the repopulated lung constructs, that provides a vascular flow path for fluid, as well as increasing barrier function within the alveolar compartment.

To understand the phenotypic changes of iPSC-ECFCs in acellular whole lung scaffolds, we processed 7,089 cells that were pre-seeded, cultured iPSC-ECFCs; and 5,591 cells from iPSC-ECFC repopulated lungs through scRNAseq. Similar to rodent primary PMECs, after culturing in acellular lung scaffolds, iPSC-ECFCs acquired markers that are related to angiogenesis, ECM/basement membrane protein synthesis, cell adhesion, and cell junctions, as compared to pre-seeded iPSC-ECFCs that had been expanded on tissue culture plastic (Figure 5G; Supplementar Table S4). We further compared the iPSC-ECFC datasets with a published human EC single-cell dataset (GEO number: GSE164829), which is comprised of over 15,000 vascular endothelial cells from 73 healthy individuals, both males and females (Schupp et al., 2020; Schupp et al., 2021). iPSC-ECFCs in repopulated lungs gained arterial-like or venous-like phenotypes. Arterial-like cells (iPSC ECFC lung Arterial) expressed canonical arterial (e.g., SERPINE2 and CXCL12) (Boulaftali et al., 2011), venous-like endothelium (iPSC ECFC lung Venous) expressed venous (e.g., IGFBP7 and NR2F2) markers (Schupp et al., 2020) (Supplementary Figure S8). We then compared pre-seeded iPSC-ECFCs, iPSC-ECFC-repopulated lungs, and human lung endothelium, in terms of the top markers of each native human EC subtype (Schupp et al., 2020). Cells in the venous cluster of the repopulated lungs (iPSC ECFC lung Venous) expressed a higher level of markers for native pulmonary venous endothelium, such as NR2F2, EPHB4 (Wang et al., 1998), and SELP (Thiriot et al., 2017) as compared to pre-seeded cells (Figure 5H). Similarly, the arterial cluster in repopulated lung expressed a higher level of SERPINE2, GJA5, CXCL12, and GJA4, than did the pre-seeded cells. Interestingly, iPSC-ECFCs in decellularized lung scaffolds also gained some recognized gCap markers, including NOSTRIN, FCN3, and ADGRF5 (Gillich et al., 2020; Schupp et al., 2020), whereas the expression of aCap markers are fairly dim, suggesting that the microenvironmental regulation of gCap and aCap phenotypes might be different. Increased expression of NOSTRIN, RGCC, CXCL12, SERPINE2, NR2F2, EPHB4, PLVAP, and COL15A1, was confirmed across multiple replicates by qRT-PCR (Supplementary Figure S9). Taken together, these data show that the iPSC-ECFCs start to shift their phenotype—at least partially—toward human lung EC phenotypes in acellular whole lung scaffolds, and become more native-like than pre-seeded cells.

### 2.5 Using Repopulated Lungs to Model Barrier Damage During Inflammation

**FIGURE 6:**
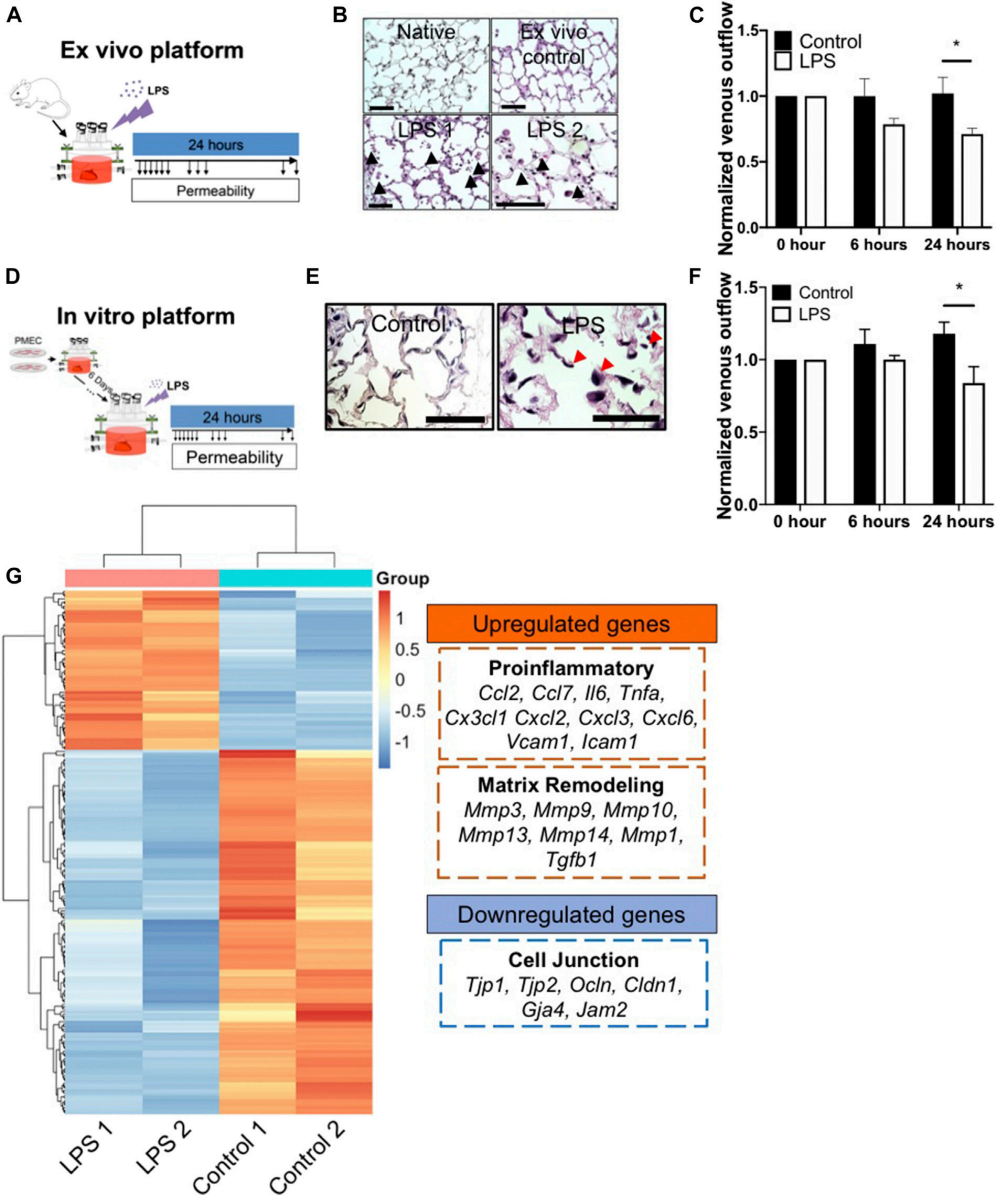
The *ex vivo* and *in vitro* platform to model LPS-induced inflammation. **(A)** Schematic of workflow for the *ex vivo* platform. **(B)** Representative H/E images of lung tissues from native, *ex vivo* control, and *ex vivo* LPS samples. Arrows indicate cell death, matrix breakdown, and interstitial puffing. Scale bar indicate 75 μm. **(C)** Venous outflows in the *ex vivo* platform with or without LPS treatment, *n* = 3. **(D)** Schematic of workflow for the *in vitro* platform. **(E)** Representative H/E images of lung tissues from control and LPS-treated repopulated lungs. Arrows indicate cell detachment and occlusion in the pulmonary vasculature. Scale bar indicate 75 μm. **(F)** Venous outflows in the repopulated lung control, and repopulated lung LPS conditions, versus time, *n* = 3. **(G)** Heatmap of top differentially (adjusted *p* < 0.05 and log2foldchange >3 or <−3) expressed genes between control and LPS-treated repopulated lungs, normalized across each gene to show up-regulation (red) and down-regulation (blue) as compared to the other group. The representative upregulated and downregulated genes after LPS treatment as compared to control without LPS were listed. * indicate *p* < 0.05. There were 3–4 replicates performed in each sample.

To evaluate the repopulated lung as a disease modeling system, we first established an *ex vivo* native lung culture platform to mimic acute lung injury, as a basis of comparison with the engineered constructs. Native lungs from adult rats were mounted into the bioreactor system and cultured with DMEM medium supplemented with 0.5% FBS and 1% antibiotics/antimycotics, and perfused at 20 ml/min via the pulmonary artery. Ten minutes after initiating arterial perfusion, we introduced 6 μg/ml LPS into the bioreactor and performed non-invasive mechanical measurements to look for evidence of barrier function and capillary leak over the ensuing 24 h (Figure 6A). As a control, we introduced a matching volume of culture medium, rather than LPS, and monitored lung mechanics over the ensuing 24 h.

After 24 h of bioreactor culture, histochemical staining of native lung samples demonstrates that the alveolar structure remains intact, resembling the morphology of freshly harvested native lungs. In contrast, LPS induced substantial cell death and visible cell detachment within the alveolar compartment (Figure 6B; Supplementary Figure S10A). Additionally, there was a markedly increased volume in the interstitial spaces at the alveolar septa in lungs treated with LPS, possibly due to the loss of microvascular alveolar barrier and influx of fluid into the lung interstitium, while the alveolar septa in the *ex vivo* control remained thin and intact (Figure 6B). Non-invasive measurements of fluid flow and barrier function revealed a marked reduction of venous outflows at 6 h after the addition of LPS to the native lungs, implying an increase in alveolar barrier permeability induced by LPS in these organs. The loss of venous flow persisted throughout the experiment, while in the control lungs, the venous outflows remained high, implying retained alveolar barrier (Figure 6C). Interestingly, capillary resistances increased over time after LPS treatment, implying a decrease of barrier function (Supplementary Figure S10B). Thus, we observed that the bioreactor system could support cellular survival, maintain the physiological barrier function for *ex vivo* native lung culture, and could model key pathological findings in response to an acute inflammatory stimulus such as LPS exposure, over a 24 h period.

To determine whether EC-repopulated lungs would exhibit similar functional changes to those of native organs, PMECs were cultured in the decellularized lung scaffolds for 6 days, followed by the introduction of a single dose of 6 μg/ml LPS into the bioreactor (Figure 6D). Within 24 h of LPS treatment, there was a substantial increase in cell death, cell detachment, and matrix degradation, as compared to control repopulated lungs not receiving LPS (Figure 6E; Supplementary Figure S10C), similar to the response observed in native lungs. H&E staining showed the cells exposed to LPS treatment appeared round and large in size. Markedly reduced numbers of patent lumens were visible 1 day after LPS exposure, whereas in control lungs the ECs displayed a flattened morphology and maintained many observable patent lumens (Figure 6E). Non-invasive mechanical measurements revealed a progressively decrease of venous outflows and an increase of capillary resistances after LPS treatment as compared to control conditions, signaling a loss of barrier function as fluid escaped from the vasculature and into the interstitium or the air sacs (Figure 6F; Supplementary Figure S10D).

Gene expression patterns from control and LPS-treated, repopulated lungs were compared using bulk RNAseq. There were a total of 2,766 genes (adjusted *p* < 0.05) differently expressed between these two conditions. The top 213 differentially expressed genes (adjusted *p* < 0.05 and Log2 fold change >3 or <−3) were extracted and clustered into a heatmap (Figure 6G). Within the top 213 differentially expressed genes, 25% of the genes were related to inflammatory response, including *Ccl2*, *Ccl7*, *Il6*, *Csf2*, *Lcn2*, *Cx3cl1*, *Cxcl2*, *Cxcl3*, *Cxcl6*, *Vcam1*, and *Icam1*. In addition, 24% of the genes were related to immune cell trafficking, suggesting that after LPS treatment, PMECs in repopulated lungs displayed a significant increase of the pro-inflammatory gene expression (Figure 6G). Additionally, matrix metalloproteases (MMPs) (e.g., *Mmp3*, *Mmp9*, *Mmp10*, and *Mmp13*) were significantly upregulated in the LPS-treated group as compared to the control group, indicating an increase in matrix degradation. Evidence of matrix degradation can also be seen in the LPS-treated repopulated lung (Figure 6E). Signals related to endothelial barrier function including *Tjp1*, *Ocln*, *Cldn1*, and *Gja4* were significantly decreased in repopulated lungs after LPS treatment compared to control conditions, consistent with the observed increase in vascular permeability. Overall, these data demonstrate that after treatment with endotoxin/LPS, PMEC-repopulated lungs displayed significantly increased pro-inflammatory signals, matrix degradation, and permeability, that simulate endothelial dysfunction during inflammation.

Therefore, these results show that the repopulated lung platform is responsive to LPS treatment in terms of increased cell death, and upregulation of inflammation-induced endothelial dysfunction signals. Combined with reduced microvascular lumen retention and an increase in permeability during culture, these findings demonstrate that LPS treatment disrupts the previously functional endothelial lining within the repopulated lungs, in ways that mimic many aspects of endothelial dysfunction during acute lung injury.

## 3 Discussion

Currently, there is no *in vitro* culture system that recapitulates structural and biological features of the pulmonary vasculature, especially the microvasculature, at a whole organ level. Whole organ decellularization provides a construct that preserves almost the entire vascular structure of a whole living organ. Here, leveraging single-cell RNA-sequencing analysis, we investigated the role of acellular whole lung scaffolds in regulating endothelial phenotypes and functions in a biomimetic bioreactor system. We found that both primary and iPSC-derived endothelium could create a degree of barrier function in lung scaffolds, and they partially gain native lung endothelial phenotypes.

To leverage this endothelilaized model platform for studying pulmonary vascular disease, we introduced LPS into the bioreactor to induce inflammation in the repopulated lung system. Similar to the reports of *in vivo* studies (Zheng et al., 2018), LPS treatment induced a marked loss of barrier function as well as dramatic histological modifications in the alveolar compartment in our *ex vivo* culture model. Analogously, in the repopulated *in vitro* platform, LPS treatment not only increased molecular signals relate to endothelial dysfunction during acute lung injury, but also induced a loss of functional endothelial lining in the pulmonary vasculature, in terms of decreased barrier function.

The development of *in vitro* systems to model human diseases is centered on recapitulating physiological properties and native cellular phenotypes. Endothelial phenotypes and functions are influenced by various *in vitro* culture conditions such as shear stress (reviewed in (Chistiakov et al., 2017)), oxygen tension (Funamoto et al., 2017), cytokine concentrations (reviewed in (Nomi et al., 2006)), ECM structure and components (Yuan et al., 2020), and substrate stiffness (reviewed in (Karki and Birukova, 2018)). In this study, we applied a biomimetic bioreactor system and optimized protocols by adding Y27632 to improve endothelial cell repopulation in the decellularized whole lung scaffolds to recapitulate *in vivo* conditions. Both primary rodent and human iPSC-derived endothelial cells display near-native appearance in the decellularized lung scaffolds. Vascular barrier can be partially reconstructed while the capillary lumens are appreciable. scRNAseq analysis reveals that after expansion on TCPS, most native signals in PMECs such as angiogenesis, cell adhesion, cell junction, and ECM synthesis are lost. Intriguingly, after only 7 days of culture in the acellular whole lung scaffolds in our biomimetic bioreactor system, many of molecular signals related to these functions could be partially restored, including increased gene expression of integrins, basement membrane proteins, receptors for growth factors, and cell-cell junction molecules, likely due to the combinational effect of cell-matrix interactions, matrix-bound growth factors, shear stress, physiological substrate stiffness, or the addition of Y27632 in our culture system.

scRNAseq has emerged as a powerful tool for identifying cell heterogeneity in native or engineered cellular systems (Hwang et al., 2018; Kumar et al., 2018; Greaney et al., 2020). In this study, we identified seven endothelial subpopulations in rat lungs, including the newly identified general capillary and aerocyte capillary endothelial cells, consistent to the recent work, including ours (Gillich et al., 2020; Travaglini et al., 2020; Schupp et al., 2021). We further leveraged established computational pipelines to compare native lung EC subtypes with *in vitro* counterparts (Greaney et al., 2020). Primary rodent PMECs that were cultured in decellularized lungs display two major clusters—arterial and venous EC, which have markedly increased expression of native markers of each EC subtype as compared to pre-seeded cells. Previous studies showed that the shear stress is responsible for arterial and venous endothelial phenotype determination (Sivarapatna et al., 2015) (Reviewed in (Baeyens et al., 2016)). In our system, the level of shear stress varies across the whole organ due to the changes of flow rate and vessel sizes, so the different regional shear stress may account for the phenotypic change into arterial or venous cells. Additionally, scRNAseq reveals marked upregulation of markers in small capillaries, more so for gCap cells rather than that for aCap cells, suggesting that ECM and shear stress could partially influence the gCap cell phenotypes, while aCap cell phenotypes may be controlled by factors that are not in our current systems, such as supporting cell populations, oxygen tension, cyclic stretch, or paracrine factors. Interestingly, we found that some cells that express mature arterial or venous markers (e.g., Eln, or Nr2f2) only attached in large vessels in the repopulated scaffolds, rather than in small vessels, suggesting a regional effect of the native matrix. However, we have also seen many cells randomly locate throughout the scaffold, suggesting that additional regional microenvironmental cues are needed to induce committed endothelial phenotypes. Human iPSC-ECFCs also start to gain some native human EC phenotypes, especially toward pulmonary venous and arterial endothelium, in decellularized lung scaffolds under current culture conditions. Although their phenotypic changes are not comparable to that in rodent primary endothelium, possibly due to species variation, it still indicates the beneficial influence of near-physiological microenvironmental cues supplied by our system.

Finally, we investigated the effects of endotoxin/LPS in our model system to simulate acute lung injury. Endothelial repopulated lungs not only display increased molecular signals that are related to pro-inflammation, matrix degradation, and decreased barrier function, but also show reduction of venous outflow and increase of capillary resistance after treatment with LPS, similar to the trends in *ex vivo* model. This result suggests that the established vascular barrier in our lung system is biologically active and their changes can be tracked at real-time.

There are still several limitations associated with the current system. First, endothelium alone cannot fully reconstitute the barrier function of native lung, due to the critical absence of differentiated type 1 alveolar epithelial cells. Therefore, changes in barrier must be viewed as relative within this system, though our studies show that barrier changes are still measurable and physiologically meaningful. Secondly, the endothelial phenotypes in repopulated lungs are still different from their native counterparts. Further development will include optimizations of oxygen tension, cyclic stretch, and inclusion of other supporting cells, as well as potentially improvements in culture medium composition to further enhance endothelial phenotype in repopulated organs.

Overall, this proof-of-principle study demonstrates that endothelium from various sources can partially simulate the native functions and phenotypes during both homeostatic and diseased states, when cultured in decellularized lung scaffolds and a biomimetic bioreactor. This tissue-engineered culture platform can be used to model vascular diseases, which paves the way for using the whole organ engineering approach for disease modeling applications.

## 4 Methods and Materials

### 4.1 PMEC Isolation and Culture

All animal procedures in this study were approved by the Yale University Institutional Animal Care and Use Committee (IACUC) and complied with the NIH Guidelines for the Care and Use of Laboratory Animals. Lung extraction and cell isolation were modified based on previous literature (Alphonse et al., 2016). For all experiments, we used the species Rattus norvegicus, strain Sprague Dawley. Postnatal day 7 pups were euthanized by intraperitoneal injection of pentobarbital (Euthasol, 150 mg/kg). Lungs were extracted from the animals and the border (∼1 mm) of all lobes were cut off and minced into small pieces. The lung pieces are then digested with collagenase/dispase (Roche) solution (0.1 U collagenase and 0.8 U dispase per mL of double-distilled (dd) water) at 37°C for 1–1.5 h under continuous agitation. After digestion, quenched the lung-digestive solution mixture with DMEM (Gibco) supplemented with 10% FBS and 1% pen/strep. After serial washes with MACS buffer (PBS with 0.1% BSA) and DMEM, the cell mixture was incubated with CD31^+^ (Roche) conjugated Dynabeads (Dynal) for 30 min at 4°C followed by magnetic selection.

The isolated cells were cultured on fibronectin-coated (2 μg/cm^2^) six well-plate at a density of 5,000 cells/cm^2^ in endothelial culture medium (Vasculife (VEGF) medium (Lifeline cell technology) supplemented with 10% FBS and 1.5% antibiotic/antimycotic (A/A)) for 7 days with daily complete medium change. Upon passaging, the contaminated (non-endothelial-like) cell colonies were removed by scraping using pipette tips followed by rinsing with pre-warmed PBS. Usually, no further beads selection is needed after this step. If many cell contaminants were seen after 7 days of culture, apply another CD31^+^ selection after trypsinization. The resulting cells were then plated on fibronectin-coated (2 μg/cm^2^) flasks at a density of 10,000 cells/cm^2^ in endothelial culture medium.

### 4.2 Immunofluorescent Staining

To characterize the phenotype of PMECs, cells were cultured on fibronectin-coated coverslips at a density of 10,000 cells/cm^2^. After 3 days of culture, cells were fixed with 2% paraformaldehyde (containing Ca^2+^/Mg^2+^) for 15 min, permeabilized with 0.1% Triton-X in PBS for 3 min, and blocked with 5% FBS PBS for 1 h at room temperature (RT). The fixed cells were then probed with primary antibodies (Table 1) including CD31, VE-Cadherin, eNOS, and vWF overnight at 4°C followed by staining with secondary antibodies (RT, 1 h) and DAPI (RT, 5 min). To detect acLDL uptake and griffonia simplicifolia lectin, fixed cells were directly incubated with Dil-acLDL or Alexa fluor 488 conjugated-lectin GSII for 2 h. All samples were then washed, mounted, and assessed with confocal microscopy (Leica SP5).

**TABLE 1:** Antibodies used for immunostaining and western blot.

Antigen	Antibody source	Catalog number
CD31	Abcam	ab28364
VE-Cadherin	ThermoFisher	36–1900
eNOS	Abcam	ab11575
vWF	Abcam	ab6994
Dil-ac-LDL	ThermoFisher	L3484
Alexa fluor 488 conjugated-lectin GSII	Vectorlabs	FL1211
ZO1	ThermoFisher	617300
Connexin 40	Abcam	ab16585
Cxcl12	ThermoFisher	PA1-29029
Prx	Novus biologicals	NBP1-89598
Aplnr	Abcam	ab214369
Sirpa	LifeSpan Biosciences	LS-B551
Coup_TFII	Abcam	ab41859
Eln	Abcam	ab21610
Ednrb	Novus biologicals	MAB4496
Fbln5	Abcam	ab202977
β1 integrin	Cell signaling	4706S
α6 integrin	Cell signaling	3750S
Acvrl1	Santa Cruz	sc-101556
Laminin	Abcam	ab14055
Car4	Abcam	ab239505
c-Kit	bioss	bs-10005R
SMA	Dako	M0851

Re-cellularized or native lung tissues were inflation-fixed with 10% formalin for 3 h, and then embedded in paraffin. De-paraffinized tissues were soaked in antigen retrieval solution (0.05% Tween-20, 1 mM EDTA, and 10 mM Tris (pH = 9)) at 75°C for 20 min, followed by blocking (10% FBS and 0.2% Triton X-100, RT, 20 min). Re-cellularized or native lung samples were stained with antibodies overnight at 4 °C followed by staining with secondary antibodies (RT, 1 h) and DAPI (RT, 5 min). All samples were then washed, mounted, and assessed with confocal microscopy (Leica SP5).

### 4.3 Transmission Electron Microscopy

Native or re-cellularized lung samples were fixed in 2.5% gluteraldehyde and 2% paraformaldehyde in 0.1 M sodium cacodylate (pH 7.4) for 30 min at RT, followed by 90 min at 4°C with continuous shaking. The fixed samples were then rinsed in 0.1 M sodium cacodylate buffer 3 times with 10 min each at RT. After fixation, the sample preparation and imaging were carried out by the Yale School of Medicine Center for Cellular and Molecular Imaging (CCMI) Electron Microscopy (EM) core facility.

### 4.4 Quantitative Real-Time Reverse Transcription-PCR

Methods for qRT-PCR are performed as per previously described (Yuan et al., 2019). In brief, total mRNA was extracted from cells, native, or re-cellularized lungs using an RNA isolation kit (Qiagen) and cDNA was synthesized (Bio-Rad). The mRNA levels of rat genes including *CD31*, *Cdh5*, *Prox1*, *Ccl21*, *Pdpn*, *Aplnr*, *Sirpa*, *Rgcc*, *Col4a1*, *Eln*, *Col15a1*, *Fbln5*, and *β-actin*, and human genes including NOSTRIN, RGCC, CXCL12, SERPINE2, NR2F2, EPHB4, PLVAP, COL15A1, and GAPDH were analyzed using SYBR green primers (purchased from Yale Oligo Synthesis Resource) and a Real-Time PCR system (Bio-Rad). The sequences of each primer are listed in Table 2.

**TABLE 2:** Primer sequences used in qRT-PCR.

Genes	Sequence	
*Gja4*	Forward	GAA​AGA​GGG​AGA​GCT​TCG​GG
	Reverse	CAT​GGT​CCA​GCC​GTA​GAG​AC
*Nr2f2*	Forward	CGG​AGG​AAC​CTG​AGC​TAC​AC
	Reverse	CTG​CCC​CTC​TGT​ACA​GCT​TC
*Aplnr*	Forward	TGT​ACG​CCA​GTG​TCT​TTT​GC
	Reverse	CTG​TTT​TCC​GGG​ATG​TCA​GT
*Sirpa*	Forward	GTC​TTC​ATC​GGT​GTG​GGT​GT
	Reverse	TTT​GTG​TCC​TGG​ATC​TGG​GT
*Rgcc*	Forward	CGC​CAC​TTC​CAC​TAT​GAG​GAG
	Reverse	TCC​GTC​GGA​GAA​TTC​AGC​TTC
*Col4a1*	Forward	AAT​ACC​AGG​ATC​CCC​TGG​AC
	Reverse	TGC​CTT​TCT​CTC​CCT​TCT​CA
*Eln*	Forward	AGC​AGC​TAA​AGC​AGC​GAA​GT
	Reverse	TGG​GAC​CCT​AAC​TCC​TGG​TC
*Col15a1*	Forward	GCC​CCC​TAC​TTC​ATC​CTC​TC
	Reverse	CAG​TAC​GGA​CCT​CCA​GGG​TA
*Fbln5*	Forward	CTC​CCC​TGC​AGA​CTT​GCT​AC
	Reverse	TCT​CAG​CAG​GGC​ACA​TAC​AG
*Vcam1*	Forward	GAA​ATG​CCA​CCC​TCA​CCT​TA
	Reverse	GAG​ATC​CAG​GGG​AGA​TGT​CA
*Icam1*	Forward	AGG​TAT​CCA​TCC​ATC​CCA​CA
	Reverse	GCC​ACA​GTT​CTC​AAA​GCA​CA
*β-actin*	Forward	GCA​GGA​GTA​CGA​TGA​GTC​CG
	Reverse	ACG​CAG​CTC​AGT​AAC​AGT​CC
NOSTRIN	Forward	TGA​GGG​ACC​CAC​TGA​CAG​AT
	Reverse	TGC​TTT​GCT​CAG​CTT​GCT​TG
RGCC	Forward	GCC​ACT​TCC​ACT​ACG​AGG​AG
	Reverse	TGA​GGA​GTG​ACA​GTG​GCA​GA
CXCL12	Forward	AGA​GCC​AAC​GTC​AAG​CAT​CT
	Reverse	CTT​TAG​CTT​CGG​GTC​AAT​GC
SERPINE2	Forward	CTT​TGA​GGA​TCC​AGC​CTC​TG
	Reverse	TGC​GTT​TCT​TTG​TGT​TCT​CG
NR2F2	Forward	TGC​CTG​TGG​TCT​CTC​TGA​TG
	Reverse	ATA​TCC​CGG​ATG​AGG​GTT​TC
COL15A1	Forward	GTT​GTC​CAC​CTA​CCG​AGC​AT
	Reverse	TTC​GCC​ATG​CTT​CAC​AGT​AG
PLVAP	Forward	GAG​CTG​GCC​ATC​AGA​AAC​TC
	Reverse	GGG​ACT​CCA​GGA​TCT​TCC​TC
EPHB4	Forward	TGA​AGA​GGT​GAT​TGG​TGC​AG
	Reverse	AGG​CCT​CGC​TCA​GAA​ACT​CAC
GAPDH	Forward	GAC​AAC​AGC​CTC​AAG​ATC​ATC​AG
	Reverse	ATG​GCA​TGG​ACT​GTG​GTC​ATG​AG

### 4.5 Western Blot

PMECs cultured on tissue culture plates were lysed in cold RIPA buffer (50 mM Tris-HCl, pH 7.4, 150 mM NaCl, 1% [v/v] Triton X-100, 0.5% [w/v] SDC, and 0.1% [w/v] sodium dodecyl sulphate (SDS)) containing 1% protease inhibitor (Sigma Aldrich, St. Louis, MO). For studies of recellularized lungs, the right middle lobes were homogenized in RIPA buffer. Protein lysate was mixed with laemmli sample buffer, reduced (DTT at 95°C 5 min), and loaded on 4–20% gradient polyacrylamide gels (Bio-Rad, Hercules, CA). Blots were run for 60 min at 120 V and the protein was transferred to PVDF membrane and blocked for 1 h at room temperature in 5% nonfat dry milk. Primary antibodies were applied overnight at 4°C in 1% nonfat dry milk in TBST buffer (0.1% Tween-20 in Tris-buffered saline) at concentrations listed in Table 1. After being rinsed with TBST, secondary antibodies were applied at a dilution of 1:1,000 for 1 h at room temperature. Protein was detected using enhanced chemiluminescence (Thermo Fischer Scientific).

### 4.6 Lung Decellularization

The decellularized lung scaffolds were prepared as per previously described (Yuan et al., 2019; Calle et al., 2011). In brief, lungs were extracted from the thorax and were perfused with antibiotics/antimycotics, and blood was cleared by additional perfusion with PBS containing heparin and sodium nitroprusside. Lungs were decellularized by serial washes with sodium deoxycholate (0.1, 0.5, and 1%) and Triton X-100 (0.0035, and 0.5%). The decellularized lungs were mounted in a sterile bioreactor and perfused with antibiotics/antimycotics for 48 h at 37°C prior to storing at 4°C. We performed H/E staining, and DNA quantification to confirm that the decellularization is complete as per previously described (Calle et al., 2016). For experiments in protein detection, we verified that the level of proteins such as integrins in decellularized lung scaffolds are under detection limit using western blot.

### 4.7 Human iPSC-Derived Endothelial Colony Forming Cells Culture

Human iPSC-derived endothelial colony forming cells (iPSC-ECFCs) were a general gift from Dr. Mervin Yoder’s group. These cells were cultured on collagen I-coated flasks (50 μg/ml) in endothelial culture medium as per previously described (Yuan et al., 2019; Prasain et al., 2014). Cells at passage 7–10 were used in the current studies.

### 4.8 PMEC and iPSC-ECFC Culture in Decellularized Lung Scaffolds

Whole decellularized lungs were mounted in a sterile bioreactor containing endothelial culture medium and perfused at 20 ml/min overnight via the pulmonary artery (Yuan et al., 2019; Engler et al., 2019; Calle et al., 2011). PMECs or human iPSC-ECFCs were suspended into culture medium supplemented with 30 μM Y27632 (Cayman) at a concentration of 250,000 cells/mL. Lungs were first seeded with 50 million PMECs from the pulmonary vein followed by seeding in the same manner the rest 50 million cells into the pulmonary artery. Thereafter, arterial perfusion was initiated with a peristaltic pump at a flow rate of 4 ml/min. This perfusion rate was maintained for 4 h and was ramped up 4 ml/min every 15 min until reaching 20 ml/min. During the ensuing 7 days of culture, half of the medium (endothelial culture medium +10 μM Y27632 (Cayman)) was replaced every day. For the lipopolysaccharide (LPS) study, ROCK inhibitor was removed by complete medium change on day 5. On day 6, a single dose of 6 μg/ml LPS (Sigma-Aldrich) was applied into the bioreactor jar in PMEC-repopulated lungs. The vascular barrier was measured and calculated by non-invasive methods as per previously described (Engler et al., 2019) for the following 24 h.

### 4.9 Measurement of Internal Pressures

All non-invasive flow and pressure measurements were based on our previous report (Engler et al., 2019). In brief, measurement of average internal pressures was accomplished by briefly shutting off flow into or out of the organ. To measure the average pressure at the end of the capillaries, flow out of the vein was briefly shut off. The average pressure in the alveoli is found in a comparable manner by briefly shutting off flow out of the trachea and monitoring the measured tracheal pressure immediately after the change. The average pressure at the start of the capillary is found in a comparable manner by briefly shutting off pulsatile perfusion to the whole organ and monitoring the measured arterial pressure immediately after the change.

### 4.10 *Ex Vivo* Lung Culture

In *ex vivo* lung culture, we used 0.25 m L/S 14 BPT tubing (Cole Parmer, PharMed) at both venous and tracheal sides. Lungs from adult rats (250–350 g) were extracted from the thorax and were perfused with antibiotics/antimycotics, and blood was cleared by additional perfusion with PBS containing heparin and sodium nitroprusside. Extracted lungs were mounted into a bioreactor system and cultured with DMEM medium supplemented with 0.5% FBS and 1% antibiotics/antimycotics and perfused at 20 ml/min via the pulmonary artery. Barrier measurements were performed as per previously described (Engler et al., 2019) for the following 24 h. To model the acute lung injury, 15 min after the start of arterial perfusion, a single dose of LPS was applied to bioreactor jar to reach a concentration of 6 μg/ml and barrier measurements were performed for the following 24 h.

### 4.11 10x scRNAseq Library Preparation, Sequencing, and Alignment

scRNAseq libraries were generated with the Chromium Single Cell 3’ assay (10x Genomics). Samples were diluted for an expected cell recovery population of 10,000 cells per lane. Libraries sequenced on the HiSeq4000 platform to a depth of 180–500 million reads per library. Downstream processing was conducted with Cellranger 3.0.2 with the default parameters. Ensembl Rnor6.0 release 95 was used to align rat data (Raredon et al., 2019).

### 4.12 scRNAseq Data Filtration, Normalization, Scaling, and Clustering

Aligned data was processed using Seurat v3.1.2 under R (v3.5.0) environment. Default parameters were used for each function unless otherwise specified. After creating Seurat objects for each sample, we filtered on percent mitochondrial genes to exclude damaged and dying cells and on the number of genes and transcripts to remove debris and doublets. The gene counts were normalized to the total counts and multiplied by 10,000 scaling factor. We used Variance Stabilizing Transformation (VST) to select highly variable genes. Top 2,000 genes within the selected genes were retained for downstream analysis. Variable genes were scaled using Seurat’s ScaleData function with regression on percent mitochondrial transcripts and cell cycle genes (Nestorowa et al., 2016), and subsequently were used for principal component analysis (PCA) to generate clusters. The numbers of clusters produced using PCA analysis are controlled by a resolution parameter with higher values giving more clusters and by the number of PCs. All cells across different samples were assigned into two-dimensional Uniform Manifold Approximation and Projection (UMAPs).

To sub-cluster postnatal day 7 endothelial cells, we first filtered, normalized, scaled the cells from whole lung sample. We then used SubsetData function in Seurat to select cell clusters that are positive for Cdh5 while negative for Ptprc, Col1a1, and Epcam to create a new postnatal day 7 lung EC Seurat object. We then re-normalized, re-scaled and re-clustered this EC object for downstream analysis.

### 4.13 Cluster Identification by Differentially Expressed Genes

The FindAllMarkers function in Seurat was applied to each sample to identify differentially expressed genes for each cluster relative to all the other clusters in the object (Greaney et al., 2020). We only chose genes that were expressed in at least 10% of cells in one of these clusters to ensure the quality of genes. These marker lists were used to apply cluster labels based on top defining genes of canonical cell types in the literature. The gene ontology analysis was performed through on-line library DAVID (Huang et al., 2009a; Huang et al., 2009b).

### 4.14 Combined

We performed Merge function in Seurat to combine different samples to allow for direct comparisons on gene expression without corrections on the batch-effect. Using top DEGs from native lung EC clusters, dot plots and violin plots were generated in merged objects using the DotPlot, and VlnPlot functions, respectively.

### 4.15 Bulk RNA Sequencing

RNA sequencing libraries were prepared by the Yale Center for Genomics Analysis using the Pair End Sequencing Prep Kit, and sequenced through NovaSeq with sequencing read length of 100 base pairs (bp). Sequenced FASTQ samples were evaluated for overrepresented sequences using FASTQC and trimmed of poly(A) sequences using Trimmomatic v 0.36 (no overrepresented adapter sequences were found in any samples). Paired sequences, all with length longer than 30 bp, were then aligned with Rsubread (Bioconductor) with the default parameters.

For RNA sequencing analysis and heatmap clustering, features were counted using Rsubread feature counts and genes were filtered to remove those with no counts across all conditions. A variance stabilizing transform was then performed on the dataset with the count matrix using the “vst” function to remove the dependence of data variance on the mean across the entire experiment. DeSeq2 (Bioconductor) was then used to determine differentially expressed genes between the two different sample comparisons. Clustered heatmaps were generated with normalized values across each gene. Ensembl Gene IDs were converted to gene symbols using biomaRt (Bioconductor).

### 4.16 Statistical Analysis

Comparisons between groups were performed with Student’s *t*-test or, for multi-group comparisons, by analysis of variance (ANOVA) followed by a post-hoc test. All analysis was performed with GraphPad Prism 7.0; values shown graphically are mean ± standard error.

## Data Availability

Publicly available datasets were analyzed in this study. This data can be found on the Gene Expression Omnibus under the accession number GSE188425.
