# Supplementary material for: A Pulmonary Vascular Model From Endothelialized Whole Organ Scaffolds
Source: Front Bioeng Biotechnol. 2021 Nov 19;9:760309. doi: 10.3389/fbioe.2021.760309 (PMC8640093; doi:10.3389/fbioe.2021.760309)
Supplement: Supplementary file 3 [file DataSheet1.PDF]

Supplemental Information

Supplemental Table 1: Top markers enriched in native lung endothelium as compared to P4 PMEC.

Supplemental Table 2: Top markers enriched in repopulated lung as compared to P4 PMEC.

Supplemental Table 3: Top markers in each endothelial subtype in native lung endothelium.

Supplemental Table 4: Top markers enriched in iPSC-ECFC repopulated lung as compared to pre-seeded iPSC-ECFC.

Supplemental Figure 1: Schematic of the key components of the lung bioreactor.

Supplemental Figure 2: Characterization of primary PMECs.

Supplemental Figure 3: Cell coverage in PMEC-repopulated lungs.

Supplemental Figure 4: Validation of integrins, ECM proteins, and angiogenesis markers in pre-seeding PMEC, and in repopulated lungs.

Supplemental Figure 5: Immunostaining of native rat lung for key markers from native cluster.

Supplemental Figure 6: scRNAseq clustering of repopulated lung and P4 PMEC.

Supplemental Figure 7: Validation of scRNAseq findings with multiple replicates.

Supplemental Figure 8: scRNAseq clustering of iPSC-ECFC lung and iPSC-ECFC.

Supplemental Figure 9: qRT-PCR determination of expression of NOSTRIN, RGCC, CXCL12, SERPINE2, NR2F2, EPHB4, PLVAP, and COL15A1 in pre-seeding iPSC-ECFC, and in iPSC-ECFC repopulated lungs.

Supplemental Figure 10: Additional characterization of ex vivo and in vitro system after LPS treatment.

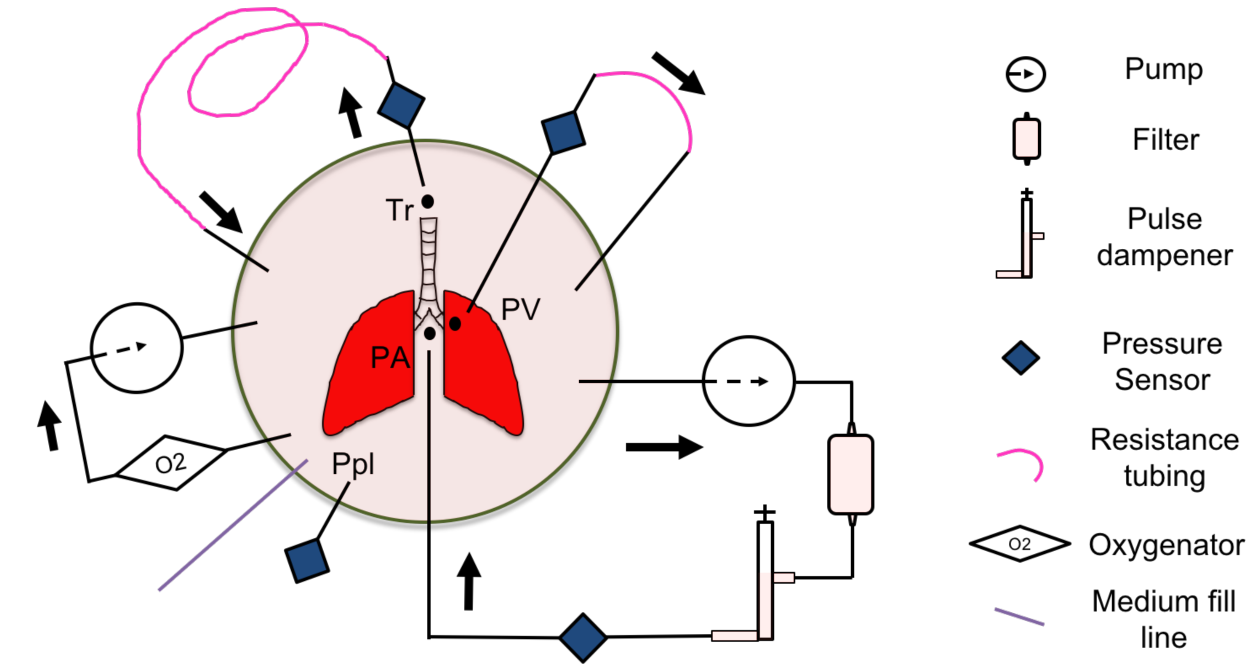

PA: Pulmonary artery; PV: Pulmonary venous; Tr: Tracheal; Ppl: Pleural pressure

Supplemental Figure 1: Schematic of the key components of the lung bioreactor. The medium was perfused from the bioreactor jar into PA and the medium outlet was coming out of the venous, tracheal and pleural ports. The pressures at pulmonary artery (PA), pulmonary vein (PV), trachea (Tr) and the medium jar (Ppl) were monitored through pressure sensors.

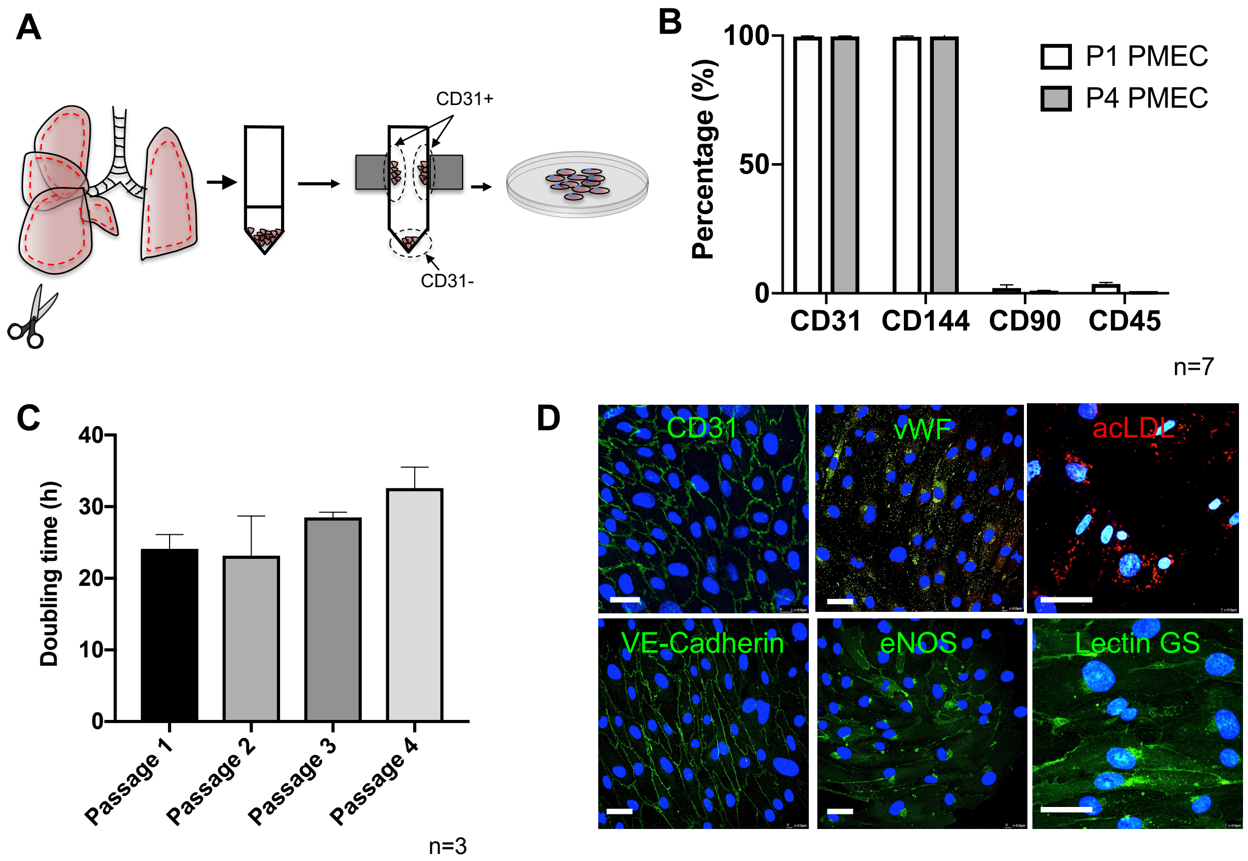

Supplemental Figure2: Characterization of primary PMECs. (A) Schematic of the workflow for endothelial cells isolation from lungs. **(B)** Flow cytometry analysis on CD31, CD144, CD90, and CD45 in P1 and P4 PMECs presented as percentage of positive cells as compared to isotype control. **(C)** Doubling times of PMECs at different passages. **(D)** Immunostaining images of CD31, vWF, acLDL uptake, VE-Cadherin, eNOS, and Lectin GS in P4 PMECs. Scale bars indicate 20 µm.

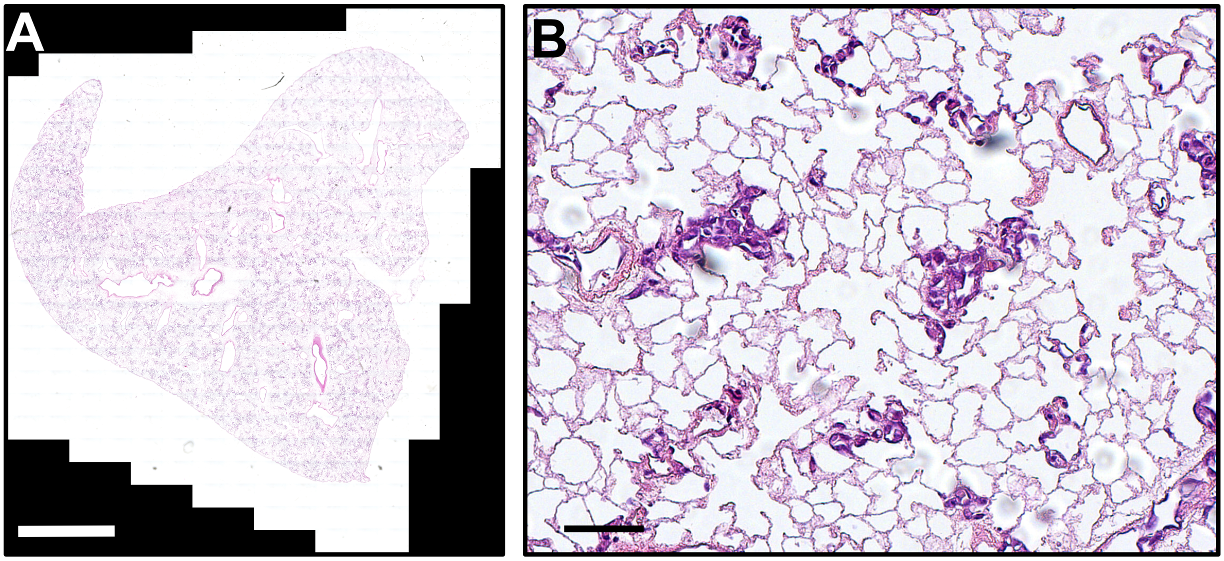

Supplemental Figure 3: Cell coverage in PMEC-repopulated lungs. (A) Representative H/E image of the whole lung lobe of PMEC-repopulated lungs after 7 days culture, scale bar 2,000 µm. **(B)** Representative H/E image of the PMEC-repopulated lungs 4 hours post-seeding, scale bar 100 µm.

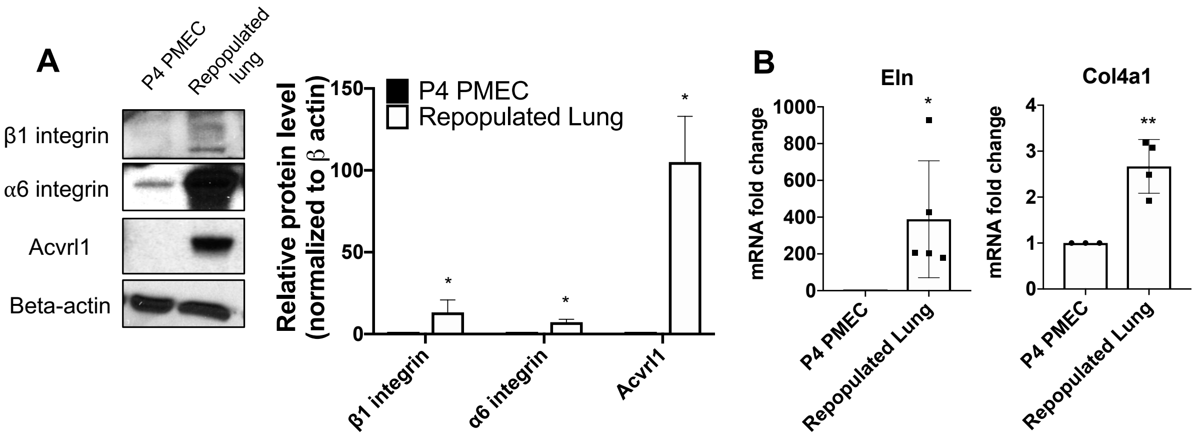

Supplemental Figure 4: Validation of integrins, ECM proteins, and angiogenesis markers in pre-seeding PMEC, and in repopulated lungs. (A) Western blot on β1 integrin, α6 integrin, Acvrl1, and β-actin in P4 PMECs and Repopulated lungs (n = 3 samples each). **(B)** Gene expression of *Eln*, and *Col4a1* in P4 PMECs and Repopulated lungs. *, and ** indicate $p < 0.05$, and $p < 0.01$, respectively. n indicates experimental replicates.

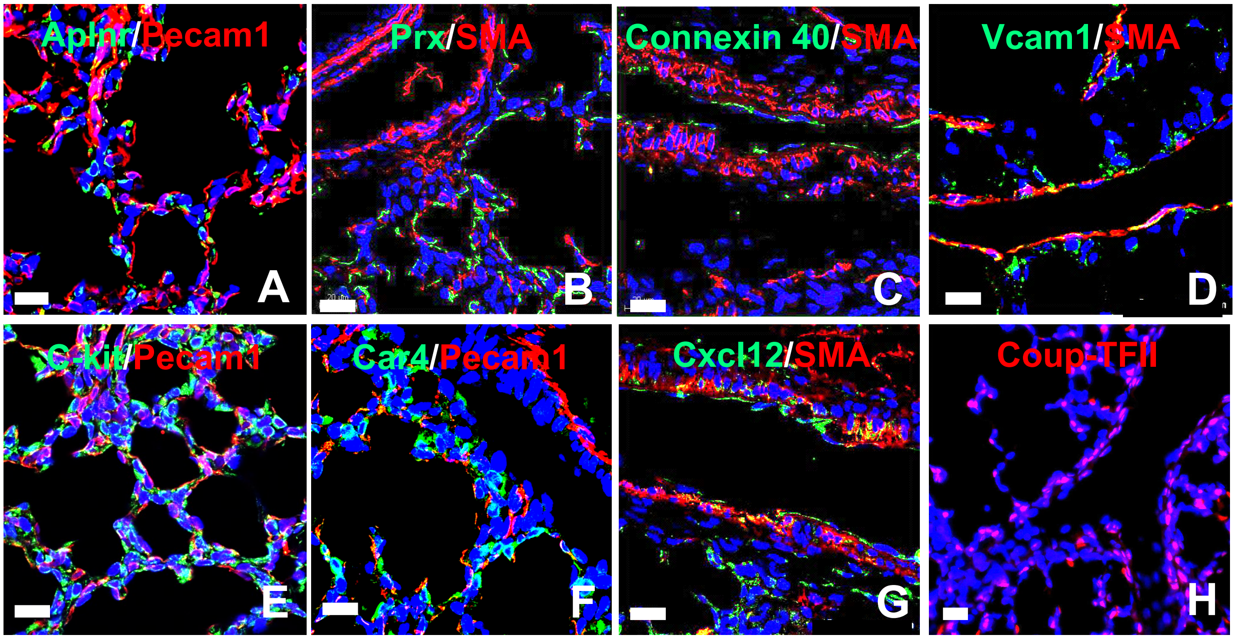

Supplemental Figure 5: Immunostaining of native rat lung for key markers from each cluster, including Connexin40 (gene name: *Gja5*) and Cxcl12 for arterial ECs; Vcam1 and Coup-TFII (gene name: *Nr2f2*) for venous ECs; Prx and Car4 for aCap ECs; Aplnr and c-kit for gCap ECs. Mesenchyme was stained by smooth muscle actin and pan-endothelium was stained by Pecam1. Nuclei were counterstained with DAPI. Scale bars indicate 20 µm.

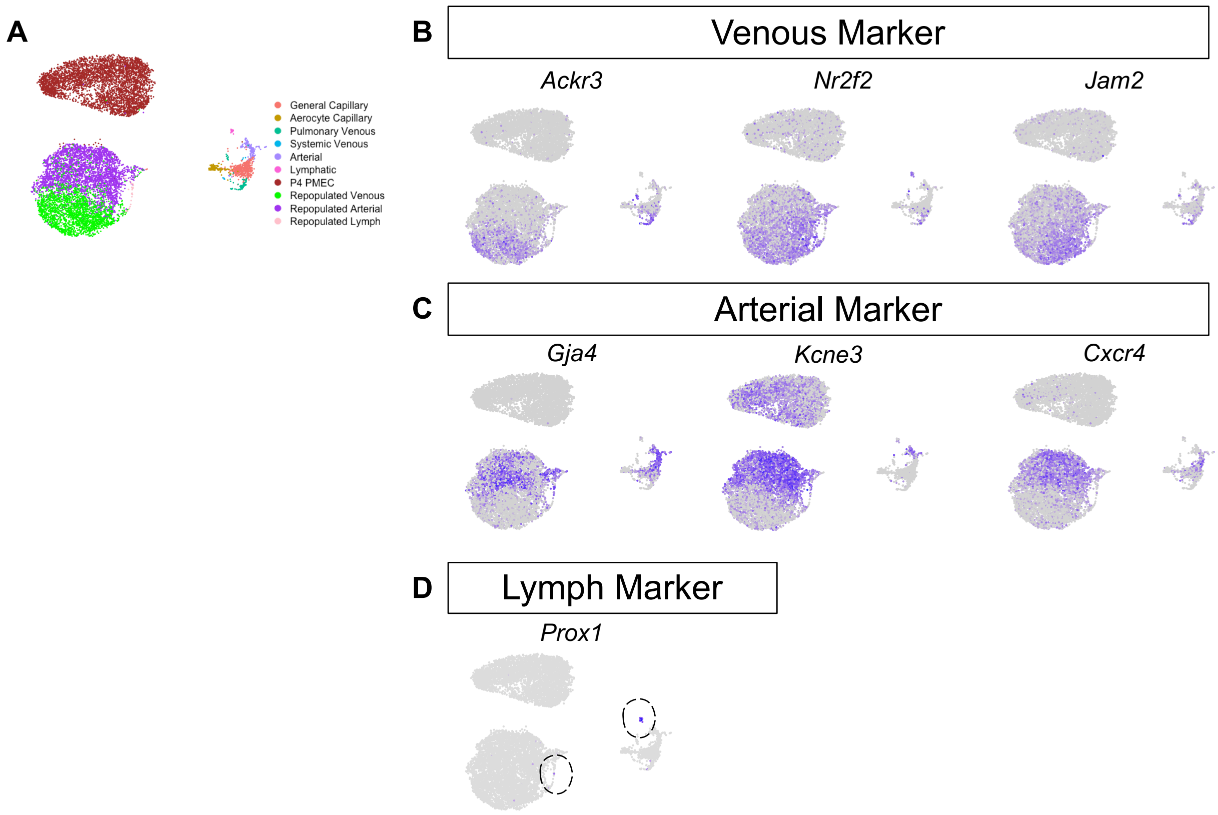

Supplemental Figure 6: scRNAseq clustering of repopulated lung and P4 PMEC. (A) UMAPplot of Merged object amongst native lung endothelium, P4 PMEC, PMEC-repopulated lung. Samples were clustered independently but merged here for aid of visualization. **(B)** FeaturePlots of representative pulmonary venous markers *Ackr3, Nr2f2,* and *Jam2* in the merged object. **(C)** FeaturePlots of representative arterial markers *Gja4, Kcne3,* and *Cxcr4* in the merged object. **(D)** FeaturePlot of representative lymph marker *Prox1* in the merged object shows that repopulated lungs have a small cluster of cells that express lymph markers. The Prox1+ cells were highlighted by dotted ovals.

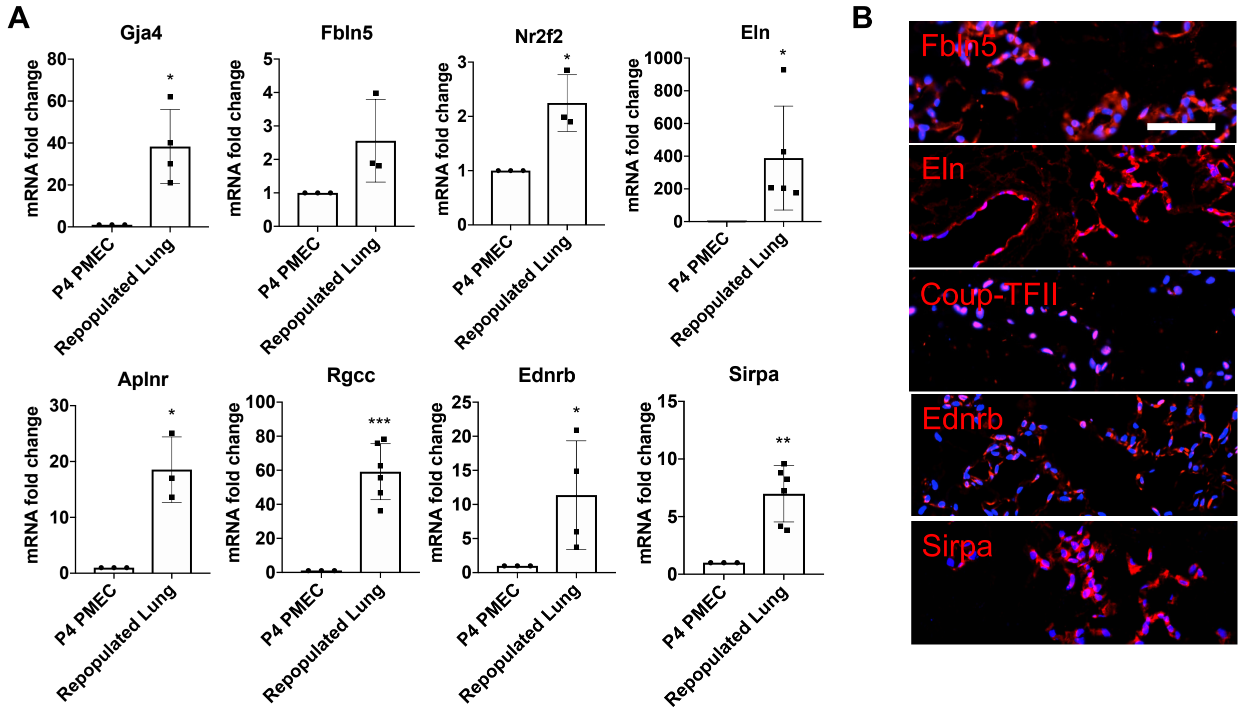

Supplemental Figure 7: Validation of scRNAseq findings with multiple replicates. (A) qRT-PCR determination of expression of *Gja4, Fbln5, Nr2f2, Eln, Aplnr, Rgcc, Sirpa,* and *Ednrb* in pre-seeding PMEC, and in repopulated lungs**.** n indicates experimental replicates. *, **, and *** indicate $p < 0.05$, $p < 0.01$, and $p < 0.001$, respectively. **(B)** Immunostaining of Fbln5, Eln, Coup-TFII, Ednrb, and Sirpa in repopulated lungs. Scale bar 20 μm.

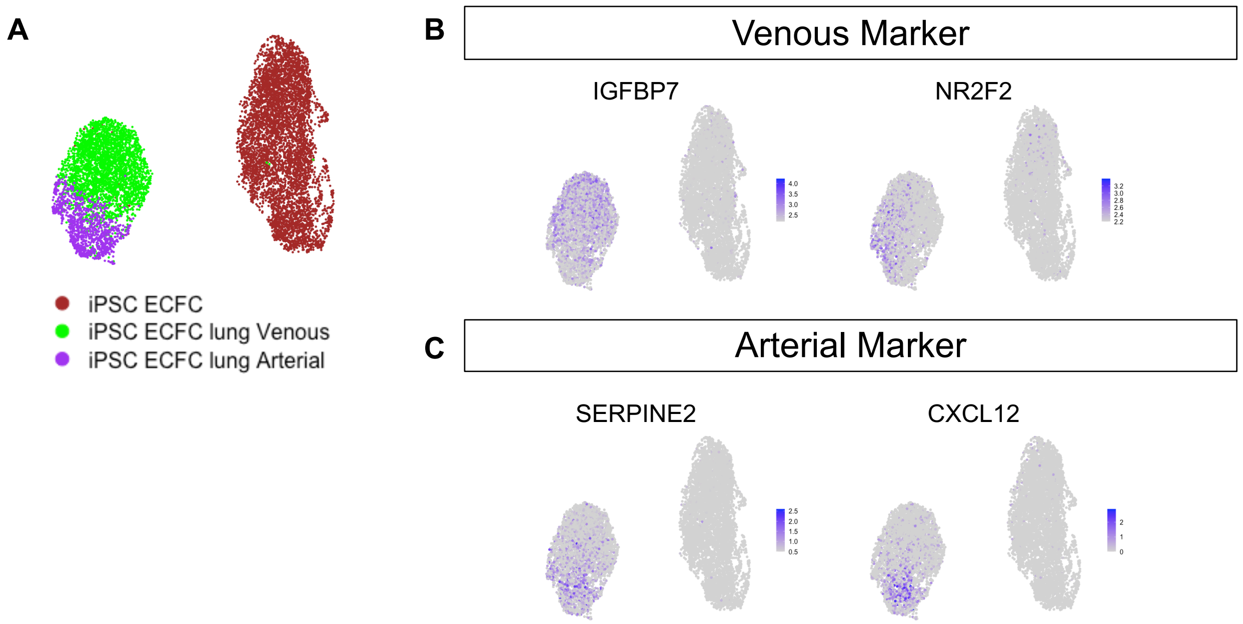

Supplemental Figure 8: scRNAseq clustering of iPSC-ECFC lung and iPSC-ECFC. (A) UMAPplot of Merged object amongst iPSC-ECFC, and iPSC-ECFC lung. Samples were clustered independently but merged here for aid of visualization. **(B)** FeaturePlots of representative human pulmonary venous markers IGFBP7, and NR2F2 in the merged object. **(C)** FeaturePlots of representative human pulmonary arterial markers SERPINE2, and CXCL12 in the merged object.

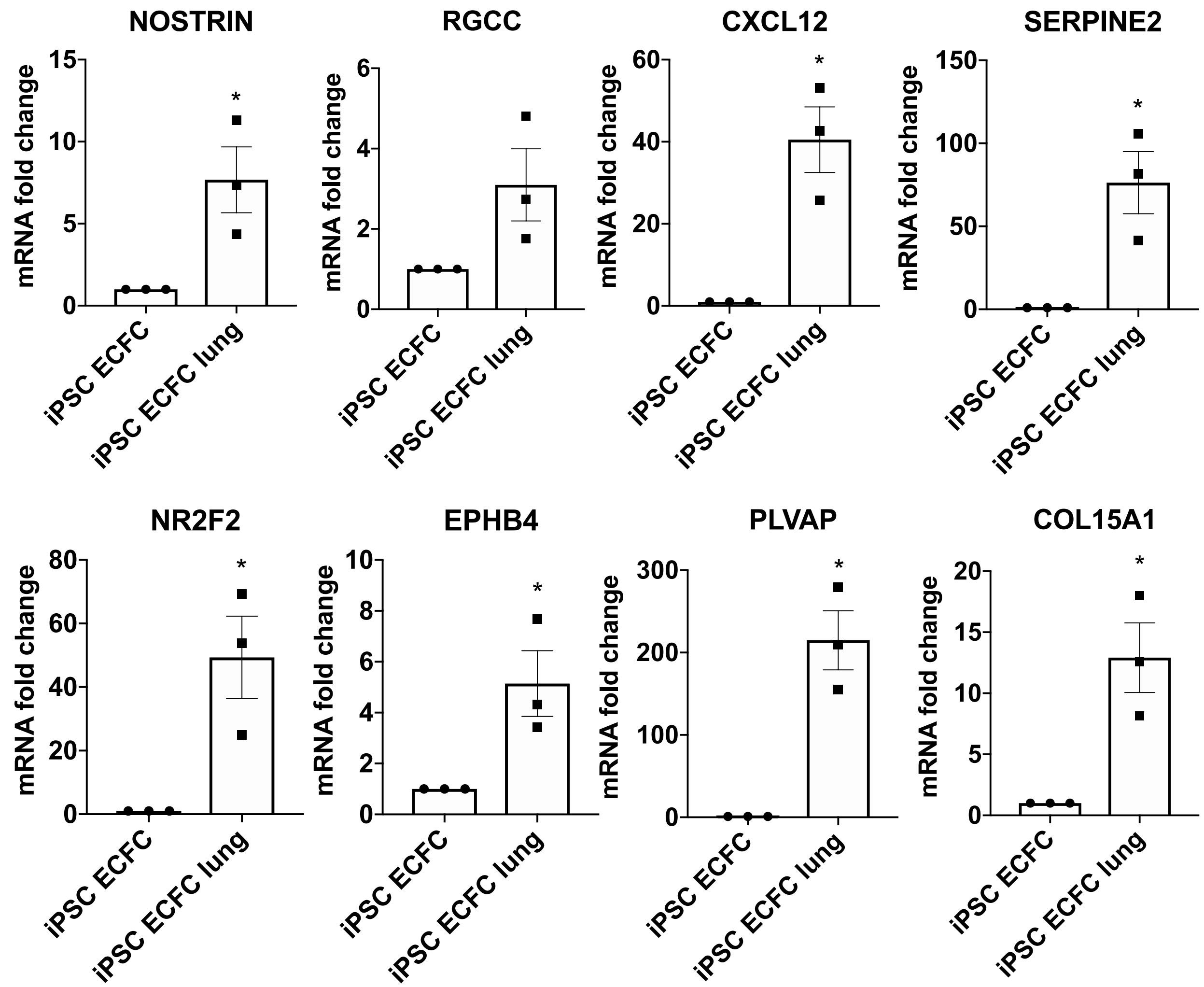

Supplemental Figure 9: qRT-PCR determination of expression of NOSTRIN, RGCC, CXCL12, SERPINE2, NR2F2, EPHB4, PLVAP, and COL15A1 in pre-seeding iPSC-ECFC, and in iPSC-ECFC repopulated lungs**.** n indicates experimental replicates. * indicates $p < 0.05$.

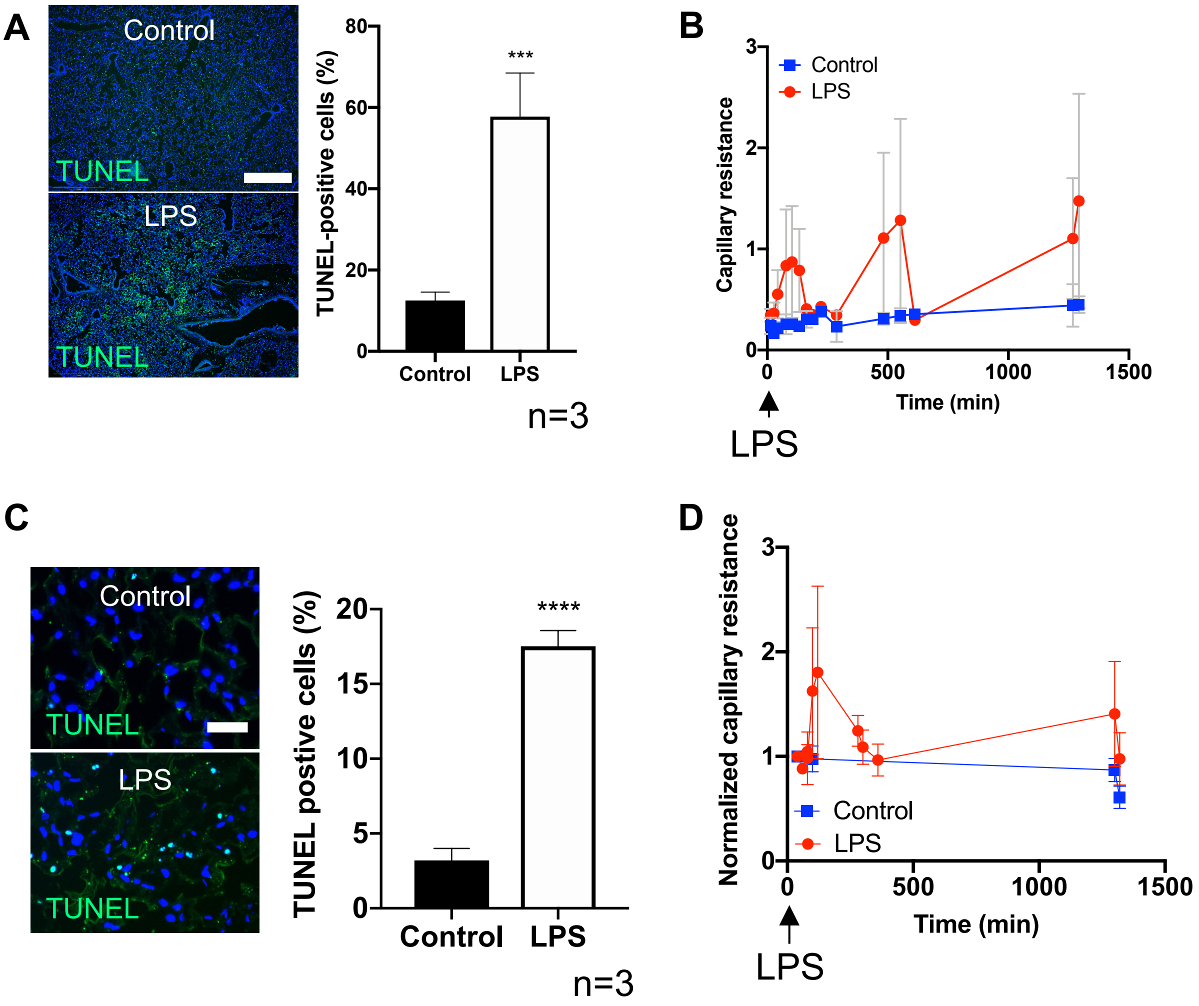

Supplemental Figure 10: Additional characterization of ex vivo and in vitro system after LPS treatment. (A) TUNEL staining of the control and LPS groups in the ex vivo platform. Scale bar indicates 750 µm. **(B)** Capillary resistances in the ex vivo platform with or without LPS treatment. **(C)** TUNEL staining of the control and LPS groups in the in vitro platform. Scale bar indicates 75 µm. **(D)** capillary resistances in the repopulated lung control, and repopulated lung LPS conditions, versus time. n indicates biological replicates. There were 3 – 4 experimental replicates used in non-invasive mechanical analyses for ex vivo and in vitro platforms.
